# In Vitro Liver Metabolism of Six Flavonoid C-Glycosides

**DOI:** 10.3390/molecules26216632

**Published:** 2021-11-01

**Authors:** Martina Tremmel, Christian Paetz, Jörg Heilmann

**Affiliations:** 1 Department of Chemistry and Pharmacy, Institute of Pharmaceutical Biology, University of Regensburg, Universitätsstraße 31, D-93053 Regensburg, Germany; martina.tremmel@ur.de; 2 Research Group Biosynthesis/NMR, Max-Planck-Institute for Chemical Ecology, Hans-Knöll-Str. 8, 07745 Jena, Germany; cpaetz@ice.mpg.de

**Keywords:** flavonoid C-glycosides, liver metabolism, S9 fraction, human liver microsomes

## Abstract

Several medical plants belonging to the genera *Passiflora*, *Viola*, and *Crataegus* accumulate flavonoid C-glycosides, which likely contribute to their efficacy. Information regarding their phase I and II metabolism in the liver are lacking. Thus, in vitro liver metabolism of orientin, isoorientin, schaftoside, isoschaftoside, vitexin, and isovitexin, all of which accumulated in *Passiflora incarnata* L., was investigated by incubation in subcellular systems with human liver microsomes and human liver S9 fraction. All metabolite profiles were comprehensively characterized using HPLC-DAD and UHPLC–MS/MS analysis. Mono-glycosylic flavones of the luteolin-type orientin and isoorientin showed a broad range of mono-glucuronidated and mono-sulfated metabolites, whereas for mono-glycosylic flavones of the apigenin-type vitexin and isovitexin, only mono-glucuronidates could be detected. For di-glycosylic flavones of the apigenin-type schaftosid and isoschaftosid, no phase I or II metabolites were identified. The main metabolite of isoorientin was isolated using solid-phase extraction and prep. HPLC-DAD and identified as isoorientin-3′-*O*-α-glucuronide by NMR analysis. A second isolated glucuronide was assigned as isoorientin 4′-*O*-α-glucuronide. These findings indicate that vitexin and isovitexin are metabolized preferentially by uridine 5′-diphospho glucuronosyltransferases (UGTs) in the liver. As only orientin and isoorientin showed mono-sulfated and mono-glucuronidated metabolites, the dihydroxy group in 3′,4′-position may be essential for additional sulfation by sulfotransferases (SULTs) in the liver. The diglycosylic flavones schaftoside and isoschaftoside are likely not accepted as substrates of the used liver enzymes under the chosen conditions.

## 1. Introduction

Flavonoids are secondary natural compounds widespread in plants and offer various health-promoting properties, such as antioxidative [1], anticancer [2], and anti-inflammatory activities [3,4]. They are mainly present as glycosides, differing in binding type and sugar moiety. Although most of them exist as *O*-glycosides where the sugar is linked to the aglycone via an *O*-glycosidic bond, several plants also accumulate C-glycosides with sugar and aglycone linked via a C-C-glycosylic bond. A prominent example is the medicinal plant *Passiflora incarnata* L. (Passifloraceae), predominantly containing flavone C-glycosides of the luteolin and apigenin types. It is traditionally used against insomnia, anxiety, and nervous stress [5,6], and flavonoids represent a minimum of 1.5% of the dried plant [7].

Compared to C-glycosides, the metabolism of flavone *O*-glycosides is comprehensively investigated. The latter are usually absorbed as aglycones after hydrolysis in the small intestine by lactase phlorizin hydrolase (LPH) [8]. Being absorbed, the aglycones are rapidly metabolized through phase I and II enzymes in the intestinal epithelium and in the liver. In detail, phase I enzymes such as cytochrome P450s (CYPs) [9,10] and phase II enzymes such as catechol-*O*-methyltransferase (COMTs) [11,12], uridine 5′-diphospho glucuronosyltransferases (UGTs) [13,14], or sulfotransferases (SULTs) [15,16] are involved. Compared to *O*-glycosides, flavone C-glycosides are more stable, because deglycosylation to their aglycones through intestinal hydrolytic enzymes is unlikely, and absorption as intact glycosides has been assumed. Consequentially, O- and C-glycosides should differ in their metabolic behavior, but metabolism of C-glycosides has barely been investigated. As flavone C-glycosides also showed significant pharmacological properties such as in vitro antidiabetic [17], hepatoprotective [18], or anticancer [19] activity and are present in several medicinal plants of the genera *Passiflora*, *Crataegus*, and *Viola*, their metabolism should be better understood.

**Figure 1:**
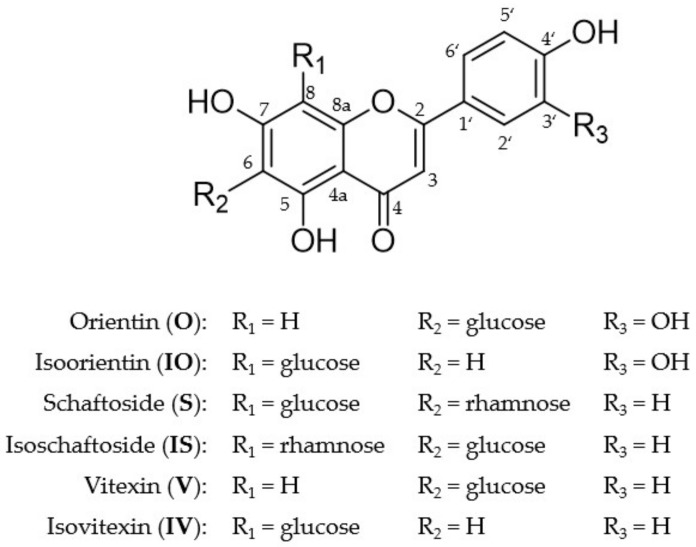
Chemical structures of the six flavone C-glycosides orientin (O), isoorientin (IO), schaftoside (S), isoschaftoside (IS), vitexin (V), and isovitexin (IV).

Isoorientin (IO; luteolin 6-C-glucoside), orientin (O; luteolin 8-C-glucoside), vitexin (V; apigenin 8-C-glucoside), isovitexin (IV; apigenin 6-C-glucoside), schaftoside (S; apigenin 6-C-glucoside-8-C-arabinoside), and isoschaftoside (IS; apigenin 8-C-glucoside-6-C-arabinoside) are six representative flavone C-glycosides accumulated in *P. incarnata* L. [20] (Figure 1). Whereas the pharmacological effects of schaftoside and isoschaftoside have not yet been examined [21], orientin and isoorientin showed various pharmacological properties [22], such as antiviral [23], antidepressant-like [24], anti-inflammatory [25,26] or antioxidative effects [27]. Vitexin exhibited antioxidative [28,29], anti-inflammatory [30], antimicrobial [31], or antiviral [32] effects, but isovitexin is poorly studied. Nevertheless, the latter seems to have similar pharmacological properties [33]. For the six above-named flavone C-glycosides, the first metabolite profiles have recently been published using a Caco-2 cell monolayer model simulating the small intestine [34]. It has been shown that they are highly metabolized by phase I and phase II enzymes. It is noteworthy that the Caco-2 cells were also able to cleave the C-C glycosylic bond [34]. Nevertheless, the metabolism of absorbed C-glycosides, especially in the liver, has not been explored either in vitro or in vivo.

**Figure 2:**
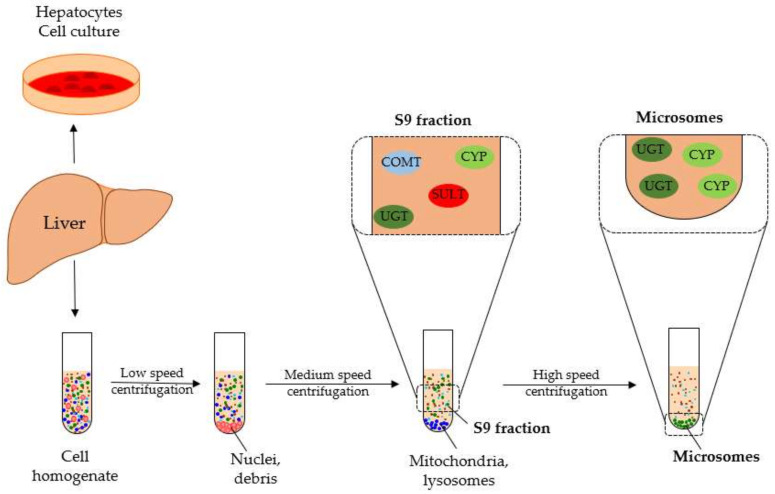
Preparation of subcellular liver fractions with enzyme profiles reliable for flavonoid metabolism of liver S9 fraction and liver microsomes; CYP: cytochrome P450, UGT: uridine 5′-diphospho-glucuronosyltransferase, SULT: sulfotransferase, COMT: catechol-*O* methyltransferase.

To simulate liver metabolism, several in vitro test systems have been established, including hepatocytes, human liver microsomes (HLM), and human liver S9 fraction (S9F). Each of these systems differ in phase I and II enzyme profiles and usability [35] (Figure 2). Although hepatocytes cover the complete enzyme profile, their handling compared to the cell-free systems is complex, and the costs are very high [36]. Liver S9 fraction contains microsomal and cytosolic fractions including important phase I and II enzymes such as SULTs, UGTs, COMTs, CYPs, *N*-acetyl transferases, and glutathione transferases [37]. In contrast, HLM include only the endoplasmic reticulum subcellular fraction of the liver, containing, most notably, CYPs and UGTs. In this study, the metabolism of six flavone C-glycosides of the apigenin and luteolin types was examined (Figure 1) using the subcellular liver fractions HLM and human S9F (Figure 2). The aim was to convey a broader understanding of in vitro and in vivo metabolism of those secondary natural compounds and to provide relevant information as scientific basis for further investigations.

## 2. Results

### 2.1. Metabolite Profiles of the Six C-Glycosides

#### 2.1.1. Metabolites Formed by HLM

**Figure 3:**
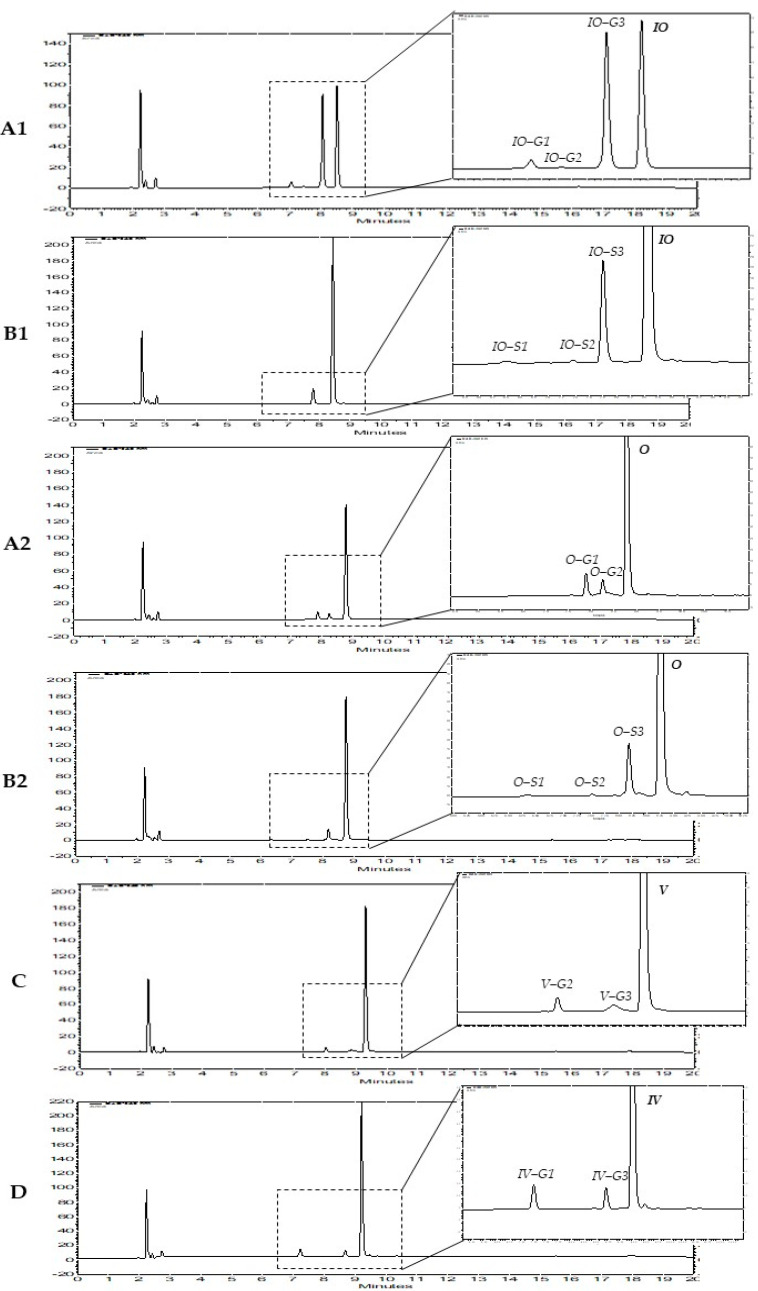
HPLC chromatograms of four flavone C-glycosides and their phase II metabolites: (**A1**) IO and its metabolites (IO-G1/G2/G3) formed by HLM, (**B1**) IO and its metabolites (IO-S1/S2/S3) formed by SULTs in liver S9 fraction, (**A2**) O and its metabolites (O-G1/G2) formed by HLM, (**B2**) O and its metabolites (O-S1/S2/S3) formed by SULTs in liver S9 fraction, (**C**) V and its main metabolites (V-G2/G3) formed by HLM, (**D**) IV and its main metabolites (IV-G1/G3) formed by HLM.

The metabolism of the six flavone C-glycosides showed that mono-glycosylic flavones orientin, isoorientin, vitexin, and isovitexin are metabolized by HLM (Figure 3), while for diglycosylic flavones schaftoside and isoschaftoside, no phase I or II metabolites were detected. The found metabolites, including LC–MS retention times, chemical notation, and related *m/z* values, are shown in Table 1.

For orientin, two metabolites, orientin-G1 and -G2 (Figure 3(A2)), with retention times 8.007 and 9.495 (Table 1), were found. Both produced [M + H]^−^ at *m/z* 623.1254 with the molecular formula C_27_H_28_O_17._ For isoorientin, three metabolites, isoorientin-G1/G2/G3, could be identified, with isoorientin-G3 being the strongly dominating compound (Figure 3(A1)). The LC–MS retention times were 6.584, 8.441, and 9.108 min (IO-G3, Table 1), respectively. The three metabolites isoorientin-G1, -G2, and -G3 produce [M + H]^−^ at *m/z* 623.1254, pointing to the molecular formula C_27_H_28_O_17_. Vitexin and isovitexin each showed three metabolites, vitexin-G1/G2/G3 and isovitexin-G1/G2/G3, respectively. For the metabolites vitexin-G1, -G2 and -G3, the LC–MS retention times 4.590, 8.072, and 10.899 min (Table 1) were determined. The retention times for the three metabolites of isovitexin were 6.813, 9.416, and 10.295 (Table 1), respectively. All six metabolites produce [M + H]^−^ at *m/z* 607.1305, corresponding to the molecular formula C_27_H_28_O_16_. In comparison to IO, all other mono-glycosylic flavones showed no strongly predominating glucuronidated metabolite (Figure 3(A2,C,D)).

The MS/MS spectra of the five metabolites of orientin and isoorientin (orientin-G1/G2 and isoorientin-G1/G2/G3) showed fragment ions at *m/z* 447, which were yielded by neutral loss of 176 Da (see Figures S1–S3, S7 and S8). For the six metabolites of vitexin and isovitexin (vitexin-G1/G2/G3 and isovitexin-G1/G2/G3), the mass spectra showed fragment ions at *m/z* 431, which were also yielded by neutral loss of 176 Da (see Figures S12–S17). The results suggest that these metabolites of orientin, isoorientin, vitexin, and isovitexin are all monoglucuronides.

It could be demonstrated that flavone C-glycosides with apigenin and luteolin as aglycone with one sugar moiety are metabolized by the UGTs in HLM, while substances with two C–C linked sugars are not metabolized at all.

#### 2.1.2. Metabolites Formed by Human S9 Fraction

The metabolism with human S9 fraction showed that, for orientin, isoorientin, vitexin, and isovitexin, the same mono-glucuronidated metabolites as described above for HLM were detected, while for schaftoside and isoschaftoside, again, no phase I or II metabolites were found. For orientin and isoorientin, three additional metabolites each, orientin-S1/S2/S3 (Figure 3(B2)) and isoorientin-S1/S2/S3 (Figure 3(B1)), respectively, were identified, with O-S3 and IO-S3 being the strongly dominating metabolites. For orientin, their LC–MS retention times were 7.692, 8.593, and 9.364 min (O-S3), respectively. All three metabolites produced [M + H]^−^ at *m/z* 527.0501, corresponding to the molecular formula C_21_H_20_O_14_S. For isoorientin, also three further metabolites, isoorientin-S1, -S2, and -S3, with retention times 6.377, 7.852, and 8.566 min could be found. All metabolites produce [M + H]^−^ at *m/z* 527.0501, pointing to the molecular formula C_21_H_20_O_14_S. The mass spectra of the six new metabolites of orientin and isoorientin (orientin-S1/S2/S3 and isoorientin-S1/S2/S3) showed fragment ions at *m/z* 447, which were yielded by neutral loss of 80 Da (see Figures S4–S6 and S9–S11). These results suggest that those six metabolites are all mono-sulfates. All obtained metabolites, including LC–MS retention times, chemical notation, and related *m/z* values, are shown in Table 1.

Thus, only the luteolin-based C-flavones orientin and isoorientin show mono-glucuronidated and mono-sulfated metabolites formed in human S9 fraction. Thus, it can be assumed that the ortho dihydroxy structure at C-3′/C-4′ in the B ring could be an essential structural feature for being accepted by the sulfotransferases expressed in human liver S9 fraction.

### 2.2. Up-Scaling and Isolation of Metabolites

By upscaling the HLM incubation assay, the isolation and NMR analysis of two main glucuronide metabolites of isoorientin were possible, whereas metabolites of other C-flavones yielded too low amounts for further structure elucidation.

#### 2.2.1. Isoorientin-3′-α-Glucuronide

**Figure 4:**
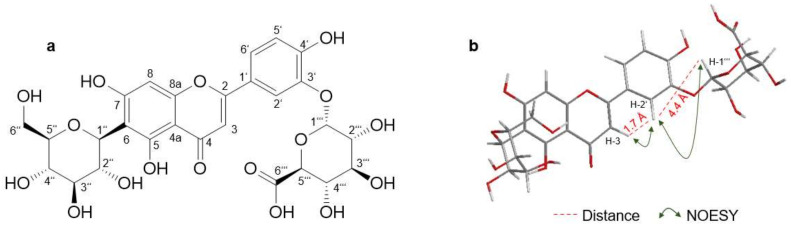
Chemical structure of isoorientin-3′-*O*-α-glucuronide (**a**) and its key NOESY correlations (**b**).

Isoorientin-3′-*O*-α-glucuronide (Isoorientin-G3, 0.4 mg, Figure 4A) was isolated as a light yellow powder with an assigned formula of C_27_H_28_O_17_ on the basis of HRESIMS (*m/z* 623.1263 [M + H]^+^, calcd. for 623.1254). NMR spectra showed resonances for 27 carbons including the 21 carbons of isoorientin, and six carbons that pertain to the glucuronic acid. The six carbons of the glucuronic acid include a carbonyl carbon (*δ_C_* 169.9 (C-6‴)) and five sp^3^ methines (*δ_H_* 5.17 (s, H-1‴), 3.36 (m, H-2‴), 3.35 (m, H-3‴), 3.42 (m, H-4‴), and 3.98 (m, H-5‴); *δ*_C_ 100.4, 72.8, 75.2, 71.2, and 74.9, respectively). The chemical shifts of the other 21 carbons are mostly comparable to those described above for isoorientin. The only remarkable deviation was noticed for position 3 (*δ_H_* 6.85 (H-3), *δ_C_* 102.9), position 2′ (*δ_H_* 7.71 (H-2′), *δ_C_* 113.8), and position 6′ (*δ_H_* 7.65 (H-6′), *δ_C_* 121.6) (Table 2 and Figures S19 and S20). The shift of those three carbons can be explained by the additional glucuronic acid on position 3′ compared to isoorientin. This could be confirmed by NOESY signals between H-3 and H-2′, and H-2′ and H-1‴, respectively (Figure 4B). In comparison, no NOESY signals between H-1‴ and H-5′ or H-6′ could be detected. The small vicinal coupling constant (*J* = 4 Hz) of the anomeric H-3‴ confirms the presence of α-glucuronic acid, which was expected due to the fact that the used cofactor uridine 5′-diphospho-glucuronic acid (UDPGA) contained glucuronic acid in its α-form.

#### 2.2.2. Isoorientin-4′-*O*-α-Glucuronide

Isoorientin-4′-*O*-α-glucuronide (Isoorientin-G1, 0.15 mg) was isolated as a light yellow powder with an assigned formula of C_27_H_28_O_17_ on the basis of HRESIMS (*m/z* 623.1264 [M + H]^+^, calcd. for 623.1254). Based on the molecular formula, it was assumed to be a structural isomer of the above described isoorientin-3′-*O*-α-glucuronide. Both structures differ only in the glucuronidation position. The glucuronyl moiety may either be located in position 7, 4′ or be attached to the sugar moiety. A connection to the hydroxy group in position 5 is unlikely due to its hydrogen bond to the ketone in position 4. Due to the small available amount, only a ^1^H NMR spectrum could be obtained, which showed several analogies to the spectrum of isoorientin-3′-*O*-α-glucuronide. The glucuronidation at position 4′ was assumed due to changes in the chemical shifts of H-5′ (*δ_H_* 6.82), H-2′ (*δ_H_* 7.68), and H-6′ (*δ_H_* 7.49) when compared to isoorientin. In contrast, the chemical shift of H-8 (*δ_H_* 6.52) was barely altered (Figure S21). The vicinal coupling constant of H-3‴ was determined as *J* = 4 Hz pointing again to the presence of an α-glucuronic acid.

## 3. Discussion

Flavonoids are of great interest because of their diverse health benefits. Unless flavone C-glycosides received lesser attention than their corresponding *O*-glycosides, they play a significant role, considering their accumulation in medicinal plants and their pharmacological activity. Although there are some studies concerning permeability and metabolism of flavone C-glycosides [38,39], information regarding their phase I and II metabolism in the liver is lacking. This study profiled the in vitro liver metabolism of six flavone C-glycosides, four mono-glycosylated, and two di-glycosylated ones in two different incubation systems. Additionally, two mono-glucuronides of isoorientin could be isolated and elucidated by NMR.

In vitro metabolism by HLM showed that the four mono-glycosylic flavone C-glycosides seem to be highly metabolized by UGTs in the chosen liver incubation systems. For IO, V, and IV three different mono-glucuronides, each could be identified, while for O, only two glucuronidated metabolites could be found (Table 1, Figure 3). S and IS are likely not substrates of the contained liver enzymes. These findings indicate that flavone C-glycosides with only one sugar moiety are highly converted by UGTs in the liver, while those with two sugar moieties are not transformed at all.

**Figure 5:**
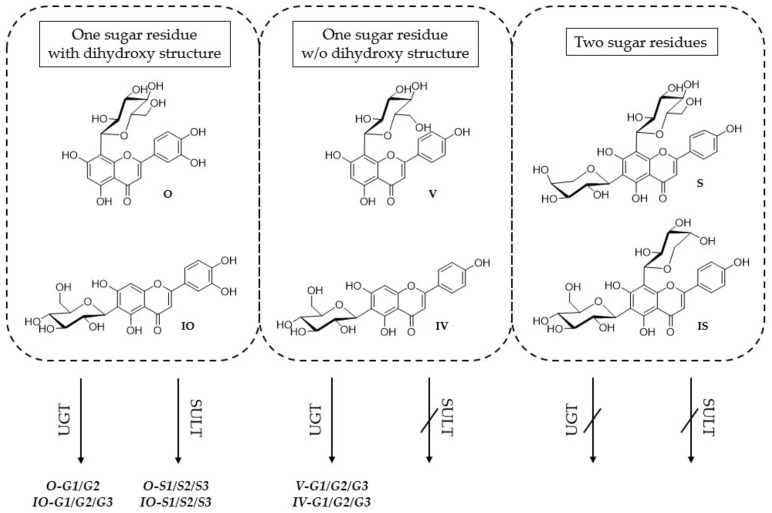
Relation between the structures of the six flavone C-glycosides, orientin (O), isoorientin (IO), vitexin (V), isovitexin (IV), schaftoside (S), and isoschaftoside (IS), and their metabolism through phase II enzymes in the liver.

As HLM do not contain SULTs, in vitro metabolism using liver S9 fraction was carried out to obtain further information concerning the conversion of the six flavone C-glycosides by UGTs and SULTs. The experiments showed no difference regarding the UGT metabolism, but mono-sulfates of orientin and isoorientin could be identified. For O, the same two, and for IO, V, and IV, the same three glucuronidated metabolites could be detected. These investigations confirm the results received using HLM as in vitro liver incubation system. Furthermore, O and IO showed three sulfated metabolites, each of which indicate that they are highly metabolized by SULTs in the liver (Table 1, Figure 3). In line, S and IS are not converted by any liver enzyme in this approach. These findings show that flavone C-glycosides with two sugar moieties might not be metabolized at all by UGTs and SULTs in the liver. Compared to V and IV, O and IO contain an ortho dihydroxy structure at C-3′/C-4′ in the B ring, which could be the essential structural feature for the conversion of C-glycosylated flavones by the SULTs expressed in the subcellular liver S9 fraction. However, the metabolism by UGTs may not depend on the dihydroxy structure in the B ring, as V, IV, O, and IO are all highly converted by UGTs (Figure 5).

As the metabolite profile of IO showed one predominating glucuronidated metabolite, the HLM approach was upscaled and worked up by solid-phase extraction and HPLC-DAD. The dominating metabolite was isolated and identified as IO-3′-*O*-α-glucuronide (IO-G3) by 2D NMR spectra (Figure 4, Table 2). When comparing the retention time of IO-G3 with the retention times of the mono-glucuronides of O, O-G2 is probably glucuronidated in 3′ position and thus is orientin-3′-*O*-α-glucuronide.

A second isolated minor mono-glucuronide of IO, IO-4′-*O*-α-glucuronide (IO-G1), was assigned by ^1^H-NMR spectroscopy. It is probably not formed in the case of O, considering the different retention time of O-G1. The glucuronides IO-G2 and O-G1 are not assignable to an exact structure due to their limited amounts. Glucuronidation may either be located at C-7 or at the sugar moiety. Glucuronidation of the hydroxy group at C-5 is more unlikely due to its hydrogen bond to the ketone at C-4. In the case of V and IV, no presumption according the glucuronidation position can be made so far, and further investigations are required.

Assessing the HPLC chromatograms in Figure 3, IO also showed one main sulfated metabolite IO-S3 using a SULT containing S9 approach. It can be postulated that according to the retention times IO-S3 is also metabolized in position 3′, but this has to be investigated in further studies. The main mono-sulfated metabolite of O is considered O-S3, but it cannot clearly be defined due to lacking assignments of O-G1 and O-G2. Nevertheless, only the conjugation position of IO-G1 and IO-G3 could be identified. Due to the small amounts of the other metabolites, their conjugation positions can only be assumed and not be proved.

Summarizing, this study offers first important knowledge of the in vitro metabolite profiles of flavone C-glycosides in the liver. As several medicinal plants accumulate flavonoid-C-glycosides, this is an important indication of which metabolites can be expected in vivo and thus can contribute to the efficacy of the plant extracts. As only subcellular systems have been used, further cellular in vitro and in vivo studies have to be performed to investigate the liver metabolism of C-glycosidated flavonoids more deeply. Further on, the structural diversity of the investigated compounds should be increased to identify structural prerequisites for different metabolic pathways and thus different metabolites and pharmacological activities.

## 4. Materials and Methods

### 4.1. Reagents and Chemicals

Orientin, isooorientin, vitexin, isovitexin, schaftoside, and isoschaftoside (>95%, reference substances) were purchased from Phytolab (Vestenbergsgreuth, Germany). Acetonitrile (LiChrosolv^®^), trifluoracetic acid (TFA), ethanol (p.a.), dimethylsulfoxide (DMSO), methanol (LiChrosolv^®^), magnesium chloride (MgCl_2_), alamethicin, glucose-6-phosphate (Glc-6-*P*), glucose-6-phosphate-dehydrogenase (Glc-6-PDH), uridine 5′-diphospho-glucuronic acid (UDPGA), β-nicotinamide adenine dinucleotide phosphate (NADP), 7-ethoxycumarin (7-EC), and adenosine 3′-phosphate 5′-phosphosulfate (PAPS) were purchased from Merck (Darmstadt, Germany). Phosphate buffered saline (PBS) was obtained by Biochrom AG (Berlin, Germany). Chromabond^®^ HR-X columns were received from Macherey-Nagel (Düren, Germany). Pooled human liver microsomes (HLM) and pooled human liver S9 fraction (S9F) were purchased from Thermo Fisher Scientific (Waltham, MA, USA). DMSO-*d_6_* was obtained from Deutero GmbH (Kastellaun, Germany).

### 4.2. Human Liver Microsome Metabolism Assay

The incubation procedure was processed and adjusted according to the literature [40]. For phase I metabolism, a NADPH regeneration system was used, consisting of MgCl_2_ (3.3 mM), Glc-6-*P* (3.3 mM), Glc-6-PDH (0.4 U/mL), NADP (1.3 mM), test substance and HLM (0.05 mg/mL) mixed in PBS. For phase II metabolism, UDPGA (2 mM) was further added as activated substrate and alamethicin (25 µg/mL) was applied as pore-forming peptide. Moreover, matrix controls, stability controls and controls without cofactors or HLM were carried out to exclude errors of the incubation system (Table 3). The tested substances O, IO, S, IS, V, and IV were dissolved in DMSO (20 mM) and again diluted in PBS to an end concentration in the incubation system of 100 µM. Additionally, 7-EC (250 µM) was used as positive control. The final volume (1 mL) was incubated at 37 °C for 3 and 24 h, respectively. All incubations were performed n = 3 on three independent days with freshly prepared solutions. The reaction was stopped by adding 1 mL ice-cold ethanol. The mixture was vortexed for 5 min and centrifuged (5 min, 14,000 rpm) afterwards. The supernatant was used for HPLC-DAD and UHPLC-MS analysis.

### 4.3. Liver S9 Fraction Metabolism Assay

The incubation procedure was composed in accordance with the above described HLM metabolism system. For phase I metabolism, a NADPH regeneration system was used, consisting of MgCl_2_ (3.3 mM), Glc-6-*P* (3.3 mM), Glc-6-PDH (0.4 U/mL), NADP (1.3 mM), test substance, and human liver S9 fraction (0.1 mg/mL) mixed in PBS. For phase II metabolism, UDPGA (2 mM) and PAPS (0.1 mM) were further added as activated substrates, and alamethicin (25 µg/mL) was applied as pore-forming peptide. Moreover, matrix controls, stability controls, and controls without cofactors or liver S9 fraction were carried out to exclude errors of the incubation system (Table 3). The test substances orientin, isoorientin, schaftoside, isoschaftoside, vitexin, and isovitexin were dissolved in DMSO (20 mM) and diluted in PBS to an end concentration in the incubation system of 100 µM. Additionally, 7-EC (250 µM) was used as a positive control. The final volume (1 mL) was incubated at 37 °C for 3 h. All incubations were performed on three independent days with freshly prepared solutions (n = 3). The reaction was stopped by adding 1 mL ice-cold ethanol. The mixture was vortexed for 5 min and centrifuged (5 min, 14,000 rpm) afterwards. The supernatant was used for HPLC-DAD and LC–MS analysis.

### 4.4. HPLC-DAD Analysis of Flavonoids and Their Glucuronides and Sulfates

The HPLC system consists of: Elite LaChrom with an L2200 autosampler, L2130 pump, L2350 column oven, L2444 DAD, and EZChrom Elite 3.1.7 software (Hitachi, Tokyo, Japan); column: Kinetex^®^ Biphenyl, 100 Å, 250 × 4.6 mm 5 µm (same material pre-column; Phenomenex, Torrance, CA, USA); injection volume 20 µL; oven temp. 25 °C; auto sampler temp.: 10 °C; detection wavelength: 340 nm; flow: 1.2 mL/min; A = H_2_O + 0.1% TFA, B = acetonitrile + 0.1% TFA; gradient: 0–2 min 10% B, 2–15 min 10% B → 70% B, 15–16 min 70% B → 10% B, 16–20 min 10% B. All samples were filtered (Phenex RC Membrane 0.2 um, Phenomenex, USA) before injection.

### 4.5. LC-MS and LC-MS/MS Analysis and Determination of Main Metabolites

UHPLC system: Agilent G4220A binary pump, G4226A HiP sampler, G1316C column comp., and G4212A DAD (Agilent Technologies, Santa Clara, CA, USA); column: Kinetex^®^ Biphenyl, 100 Å, 100 × 2.1 mm, 1.7 µm (Phenomenex, Torrance, CA, USA); injection volume: 0.2 µL; oven temp.: 25 °C; auto sampler temp.: 25 °C; detection wavelength: 190–640 nm; flow: 0.4 mL/min; A = H_2_O + 0.1% formic acid (FA), B = acetonitrile + 0.1% FA; gradient: 0–2 min 5% B, 2–1 min 5% B → 20% B, 15–16 min 20% B → 98% B, 16–17 min 98% B, 17–17.1 min 98% B → 5% B, 17.1–18.1 min 5% B. All samples were filtered (Phenex RC Membrane 0.2 um, Phenomenex, USA) before injection.

MS system: Agilent MS Q-TOF 6540 UHD, ion source: AJS ESI (Agilent Technologies, Santa Clara, USA); detection range: 80–1400 *m/z*; ion-polarity: negative; scan-rate: 4.00 spectra/s; gas-temp.: 300 °C; gas-flow: 8 L/min; nebulizer: 35 psi; sheath-gas-temp.: 300 °C; sheath-gas-flow: 10 L/min; for MS/MS experiments, the collision gas was nitrogen and the collision energy was 10, 20 or 40 eV.

The evaluation of all MS data was performed with MassHunter Qualitative Analysis B.08.00 (Agilent Technologies, USA). The masses of the resulting signals were compared with the exact mass of possible phase I and phase II metabolites. MS chromatogram peak filter: ≥100 counts, ≤10 ppm, molecular feature extraction *m*/*z* range = 60–1400, ionic polarity = negative, allowed ions = [M-H]^−^.

### 4.6. Isolation of Metabolites

#### 4.6.1. Optimized and Upscaled HLM Metabolism Assay for Metabolite Isolation

The incubation procedure was performed as described above. The concentration of HLM and UDPGA was doubled to 0.1 mg/mL and 4 mM, respectively. The final volume (14 mL) was incubated at 37 °C for 24 h. Afterwards, the reactions mixture was purified by solid phase extraction.

#### 4.6.2. Solid Phase Extraction

The column (Macherey-Nagel Chromabond^®^ HR-X) was prewashed with two column volumes (3 mL) of methanol and afterwards equilibrated with two column volumes of water. Then, the metabolite solution was gravity-loaded, and the column was subsequently washed with water. The samples were eluted with 2 mL of methanol and evaporated to dryness using N_2_ gas.

#### 4.6.3. Isolation via Analytical HPLC

For the isolation, the same HPLC system was used as described above. Column: Kinetex^®^ Biphenyl, 100 Å, 250–4.6 mm (5 µm, same material pre-column; Phenomenex, Torrance, CA, USA); Injection volume 10 µL; oven temp.: 25 °C; auto sampler temp.: 10 °C; detection wavelength: 340 nm; flow: 1.2 mL/min; A = H_2_O + 0.1% TFA, B = acetonitrile + 0.1% TFA; gradient: 0–2 min 10% B, 2–15 min 10% B → 70% B, 15–16 min 70% B → 10% B, 16–20 min 10% B. The peaks of the metabolites (retention times: 6.85 min for IO-G1, 7.86 min for IO-G3 and 8.4 min for IO) were collected separately and subsequently purified by another solid phase extraction. Additionally, 0.15 mg of IO-G1 and 0.4 mg of IO-G3 could be observed as purified metabolites.

#### 4.6.4. NMR Spectroscopy

Samples were dissolved in DMSO-*d_6_* and measured at 298 K. Chemical shifts were referenced to the residual solvent signals for DMSO-*d_6_* at *δ*_H_ 2.50 and *δ*_C_ 39.51.1D-^1^H, 1D-^13^C as well as 2D-^1^H,^13^C HSQC, ^1^H,^13^C HMBC, ^1^H,^1^H COSY, and ^1^H,^1^H ROESY NMR experiments were conducted on a Bruker AVANCE III 600 and a Bruker AVANCE III HD 700 MHz spectrometer, respectively. The latter was equipped with a TCI H-C/N-D 1.7 mm micro-cryo-probe and a cryo-platform. Data analysis and spectrometer control were accomplished using Bruker TopSpin 3.5.b.91 pl 7 (all Bruker BioSpin GmbH, Rheinstetten, Germany). Standard pulse programs as implemented in Bruker TopSpin were used.

## 5. Conclusions

The present study demonstrates that V and IV, mono-glycosoylic flavonoes of the apigenin-type, are converted preferentially by UGTs in the liver, whereas O and IO, mono-glycosylic flavones of the luteolin-type, are metabolized by both UGTs and SULTs. For di-glycosylic flavones of the apigenin type, schaftosid and isoschaftosid, no phase I or II metabolites were identified. Furthermore, two mono-glucuronidated metabolites of IO were isolated and identified as isoorientin-3′-*O*-α-glucuronide and isoorientin-4′-*O*-α-glucuronide. As only O and IO showed glucuronidated as well as sulfated metabolites, the dihydroxy group in 3′/4′-position may be essential for additional sulfation by SULTs in the liver. In addition, these findings indicate that two sugar moieties are a restriction for metabolization under the chosen conditions.

## Figures and Tables

**Table 1:** IO, O, V, IV, and their metabolites formed by human liver microsomes and liver S9 fraction analyzed by LC–MS. HLM: human liver microsomes; S9F: liver S9 fraction; G: glucuronide; S: sulfate.

Compound	Liver Fraction	RT [min]	*m/z* [M-H]^−^ Found	*m/z* [M-H]^−^ Calculated	M Formula
*Isoorientin*	HLM, S9F	9.625	447.0939	447.0933	C_21_H_20_O_11_
*Isoorientin-G1*	HLM, S9F	6.584	623.1265	623.1254	C_27_H_28_O_17_
*Isoorientin-G2*	HLM, S9F	8.441	623.1258	623.1254	C_27_H_28_O_17_
*Isoorientin-G3*	HLM, S9F	9.108	623.1263	623.1254	C_27_H_28_O_17_
*Isoorientin-OH1*	HLM, S9F	7.498	463.0877	463.0882	C_21_H_20_O_12_
*Isoorientin-S1*	S9F	6.377	527.0501	527.0501	C_21_H_20_O_14_S
*Isoorientin-S2*	S9F	7.852	527.0503	527.0501	C_21_H_20_O_14_S
*Isoorientin-S3*	S9F	8.566	527.0509	527.0501	C_21_H_20_O_14_S
*Orientin*	HLM, S9F	10.229	447.0934	447.0933	C_21_H_20_O_11_
*Orientin-G1*	HLM, S9F	8.007	623.1247	623.1254	C_27_H_28_O_17_
*Orientin-G2*	HLM, S9F	9.495	623.1249	623.1254	C_27_H_28_O_17_
*Orientin-S1*	S9F	7.692	527.0498	527.0501	C_21_H_20_O_14_S
*Orientin-S2*	S9F	8.593	527.0504	527.0501	C_21_H_20_O_14_S
*Orientin-S3*	S9F	9.364	527.0510	527.0501	C_21_H_20_O_14_S
*Vitexin*	HLM, S9F	11.525	431.0989	431.0984	C_21_H_20_O_10_
*Vitexin-G1*	HLM, S9F	4.590	607.1304	607.1305	C_27_H_28_O_16_
*Vitexin-G2*	HLM, S9F	8.072	607.1312	607.1305	C_27_H_28_O_16_
*Vitexin-G3*	HLM, S9F	10.899	607.1307	607.1305	C_27_H_28_O_16_
*Isovitexin*	HLM, S9F	11.509	431.0992	431.0984	C_21_H_20_O_10_
*Isovitexin-G1*	HLM, S9F	6.813	607.1317	607.1305	C_27_H_28_O_16_
*Isovitexin-G2*	HLM, S9F	9.416	607.1306	607.1305	C_27_H_28_O_16_
*Isovitexin-G3*	HLM, S9F	10.295	607.1313	607.1305	C_27_H_28_O_16_

**Table 2:** ^1^H and ^13^C NMR data (700 and 175 MHz, respectively; DMSO-d_6_, *δ* in ppm, *J* in Hz) for isoorientin and isoorientin-3′-*O*-α-glucuronide (s singlet, d doublet, br broad, m multiplet).

No.	Isoorientin		Isoorientin-3′-*O*-α-glucuronide	
	*δ_H_*	*δ_C_*	*δ_H_*	*δ_C_*
2		163.7		162.9
3	6.67 (1H, s)	102.5	6.85 (1H, s)	102.9
4		181.8		181.8
4a		103.4		103.1
5		159.9		160.4
6		108.5		108.5
7		163.2		163.1
8	6.48 (1H, s)	93.3	6.55 (1H, s)	93.6
8a		156.0		156.0
1′		121.3		121.3
2′	7.40 (1H, s)	113.2	7.71 (1H, s)	113.8
3′		145.3		145.0
4′		149.3		150.6
5′	6.89 (1H, d, 8.1)	115.9	6.99 (1H, d, 8.2)	116.4
6′	7.42 (1H, dd, 2.0, 8.1)	118.8	7.65 (1H, dd, 2.0, 8.2)	121.6
1″	4.58 (1H, d, 9.8)	72.9	4.59 (1H, d, 9.5)	72.8
2″	4.04 ^1^ (1H, m)	70.0	4.02^1^ (1H, m)	70.1
3″	3.19 (1H, dd, 9.0, 9.0)	78.8	3.20 (1H, m)	78.6
4″	3.12 (1H, dd, 9.0, 9.0)	70.5	3.13 (1H, m)	70.3
5″	3.16 (1H, m)	81.4	3.17 (1H, m)	81.4
6″	3.40 (1H, m) 3.68 (1H, bd, 11.0)	61.3	3.41 (1H, m) 3.68 (1H, bd, 11.0)	61.3
1‴			5.17 (1H, d, 4.0)	100.4
2‴			3.36 (1H, m)	72.8
3‴			3.35 (1H, m)	75.2
4‴			3.42 (1H, m)	71.2
5‴			3.98 (1H, m)	74.9
6‴				169.9

^1^ overlapped signal.

**Table 3:** Pipetting scheme of the human liver microsome and the human liver S9 fraction metabolism assay. HLM: human liver microsomes, S9F: human liver S9 fraction; matrix: matrix control, stab: stability control, w/o cof: control without cofactors, w/o HLM: control without HLM, w/o S9F: control without S9F, Ph I + II: phase I and II metabolism; PBS: phosphate-buffered saline; Glc-6-*P*: glucose-6-phosphate; Glc-6-PDH: glucose-6-phosphate dehydrogenase; NADP: β-nicotinamide adenine dinucleotide phosphate; UDPGA: uridine 5′-diphospho-glucuronic acid; PAPS: adenosine 3′-phosphate 5′-phosphosulfate.

Reagent	HLM					S9F				
	Matrix	Stab	w/o cof	w/o HLM	Ph I + II	Matrix	Stab	w/o cof	w/o S9F	Ph I + II
PBS	X	X	X	X	X	X	X	X	X	X
Glc-6-*P*	X		X	X	X	X		X	X	X
Glc-6-PDH	X		X	X	X	X		X	X	X
alamethicin	X		X	X	X	X		X	X	X
[substance]		X	X	X	X		X	X	X	X
HLM	X		X		X					
S9F						X		X		X
NADP	X			X	X	X			X	X
UDPGA	X			X	X	X			X	X
PAPS						X			X	X

## Data Availability

Sample of isoorientin-3′-α-glucuronide is available from J.H.

## Supplementary Materials

The following are available online: Figure S1: MS/MS spectrum of the extracted mass of isoorientin-G1 and its retention time with loss of dehydrated (-18 u) glucuronic acid (GlucA). Figure S2: MS/MS spectrum of the extracted mass of isoorientin-G2 and its retention time with loss of dehydrated (-18 u) glucuronic acid (GlucA). Figure S3: MS/MS spectrum of the extracted mass of isoorientin-G3 and its retention time with loss of dehydrated (-18 u) glucuronic acid (GlucA). Figure S4: MS/MS spectrum of the extracted mass of isoorientin-S1 and its retention time with neutral loss of SO_3_. Figure S5: MS/MS spectrum of the extracted mass of isoorientin-S2 and its retention time with neutral loss of SO_3_. Figure S6: MS/MS spectrum of the extracted mass of isoorientin-S3 and its retention time with neutral loss of SO_3_. Figure S7: MS/MS spectrum of the extracted mass of orientin-G1 and its retention time with loss of dehydrated (-18 u) glucuronic acid (GlucA). Figure S8: MS/MS spectrum of the extracted mass of orientin-G2 and its retention time with loss of dehydrated (-18 u) glucuronic acid (GlucA). Figure S9: MS/MS spectrum of the extracted mass of orientin-S1 and its retention time with neutral loss of SO_3_. Figure S10: MS/MS spectrum of the extracted mass of orientin-S2 and its retention time with neutral loss of SO_3_. Figure S11: MS/MS spectrum of the extracted mass of orientin-S3 and its retention time with neutral loss of SO_3_. Figure S12: MS/MS spectrum of the extracted mass of vitexin-G1 and its retention time with loss of dehydrated (-18 u) glucuronic acid (GlucA). Figure S13: MS/MS spectrum of the extracted mass of vitexin-G2 and its retention time with loss of dehydrated (-18 u) glucuronic acid (GlucA). Figure S14: MS/MS spectrum of the extracted mass of vitexin-G3 and its retention time with loss of dehydrated (-18 u) glucuronic acid (GlucA). Figure S15: MS/MS spectrum of the extracted mass of isovitexin-G1 and its retention time with loss of dehydrated (-18 u) glucuronic acid (GlucA). Figure S16: MS/MS spectrum of the extracted mass of isovitexin-G2 and its retention time with loss of dehydrated (-18 u) glucuronic acid (GlucA). Figure S17: MS/MS spectrum of the extracted mass of isovitexin-G3 and its retention time with loss of dehydrated (-18 u) glucuronic acid (GlucA). Figure S18: ^1^H-NMR spectrum of isoorientin in DMSO-*d_6_* at 298 K, 700 MHz. Figure S19: ^1^H-NMR spectrum of isoorientin-3′-*O*-α-glucuronide in DMSO-*d_6_* at 298 K, 700 MHz. Figure S20: Expanded ^1^H NMR spectrum of isoorientin-3′-*O*-α-glucuronide with coupling constants of H-1‴ and H-1″. Figure S21: ^1^H-NMR spectrum of isoorientin-4′-*O*-α-glucuronide in DMSO-*d_6_* at 298 K, 700 MHz.
