# Supplementary material for: In Vitro Liver Metabolism of Six Flavonoid C-Glycosides
Source: Molecules. 2021 Nov 1;26(21):6632. doi: 10.3390/molecules26216632 (PMC8587677; doi:10.3390/molecules26216632)
Supplement: Supplementary file 1 [file molecules-26-06632-s001.zip › molecules-1396343-supplementary.pdf]

Supplementary Materials

In Vitro Liver Metabolism of Six Flavonoid C-Glycosides

Martina Tremmel [1], Christian Paetz [2] and Jörg Heilmann [1,*]

[1] Institute of Pharmaceutical Biology, Department of Chemistry and Pharmacy, University of Regensburg, Universitätsstr. 31, D-93053 Regensburg, Germany; martina.tremmel@ur.de
[2] Max-Planck-Institute for Chemical Ecology, Research Group Biosynthesis/NMR, Hans-Knöll-Str. 8, 07745 Jena, Germany, cpaetz@ice.mpg.de
* Correspondence: joerg.heilmann@chemie.uni-regensburg.de; Tel.: +49 941-943-4759

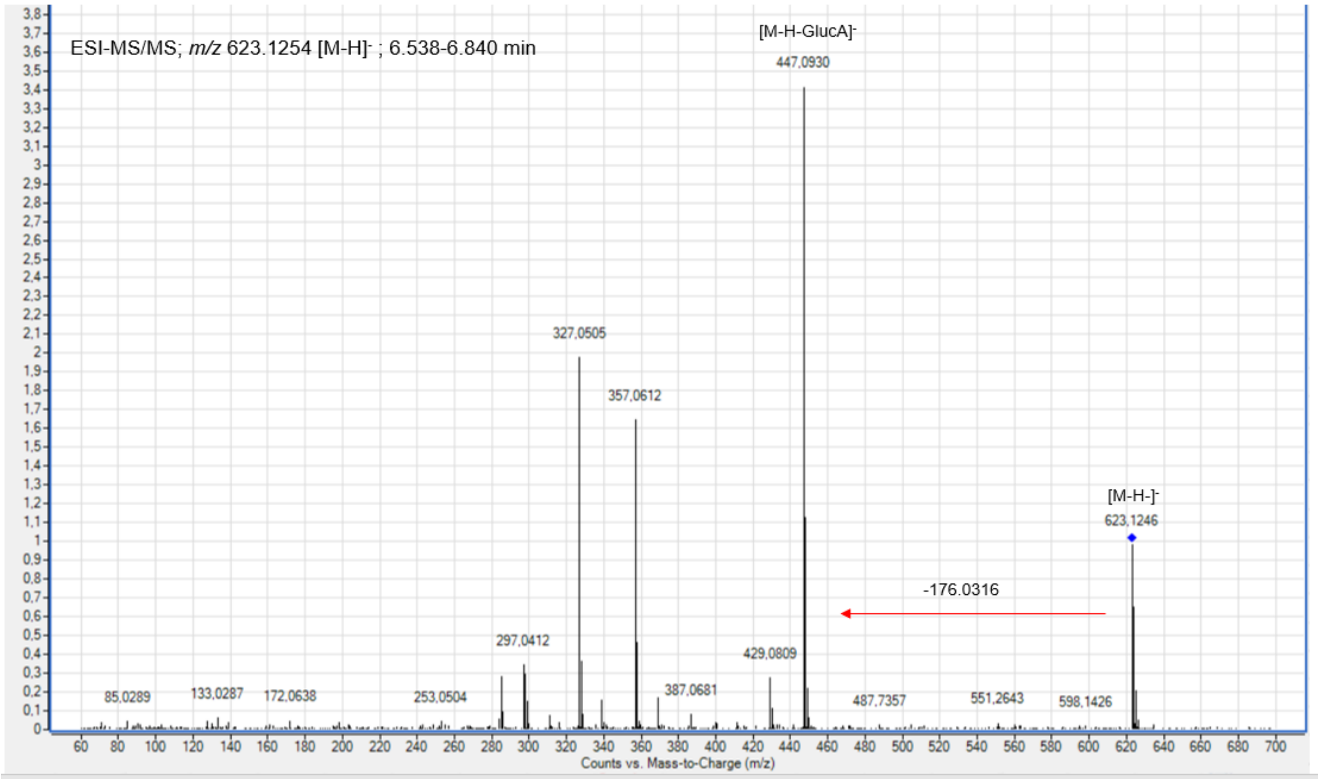

Figure S1. MS/MS spectrum of the extracted mass of isoorientin-G1 and its retention time with loss of dehydrated (-18 u) glucuronic acid (GlucA).

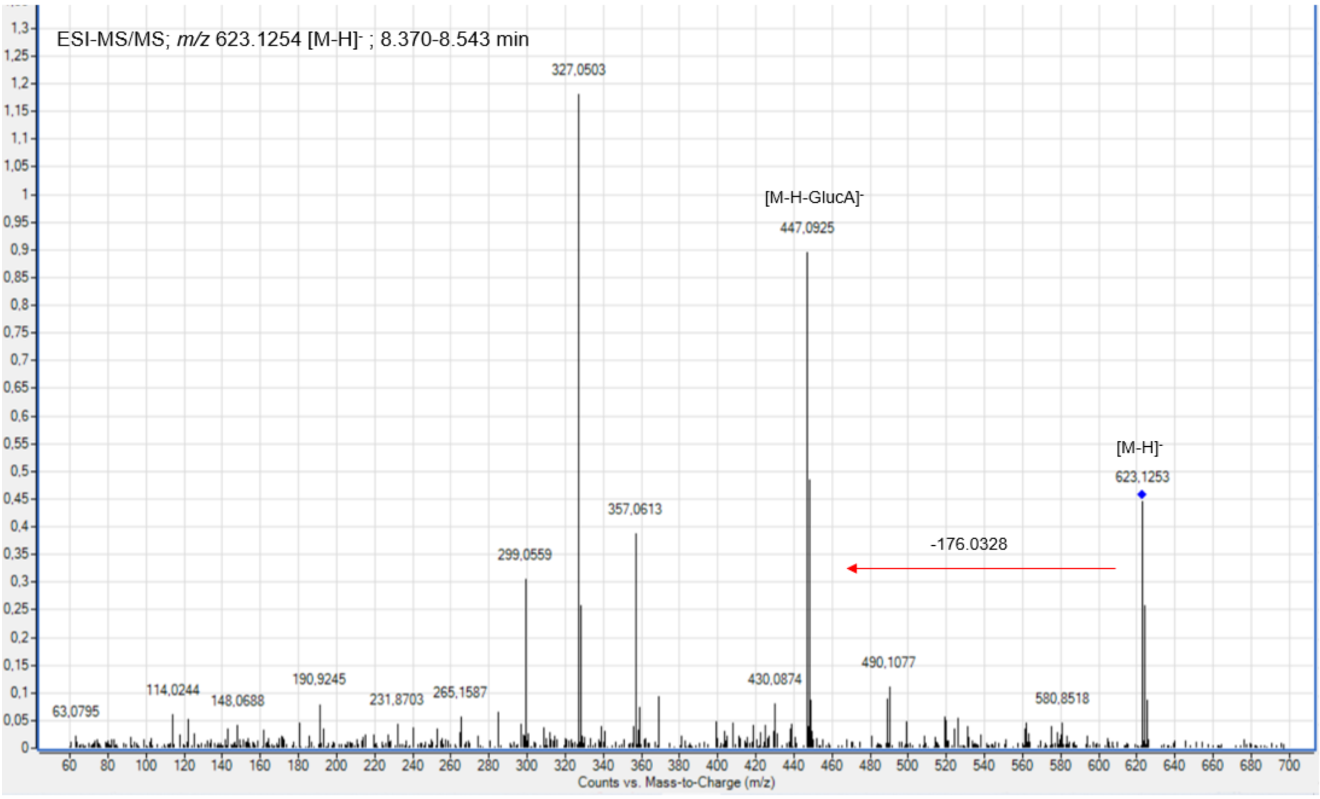

Figure S2. MS/MS spectrum of the extracted mass of isoorientin-G2 and its retention time with loss of dehydrated (-18 u) glucuronic acid (GlucA).

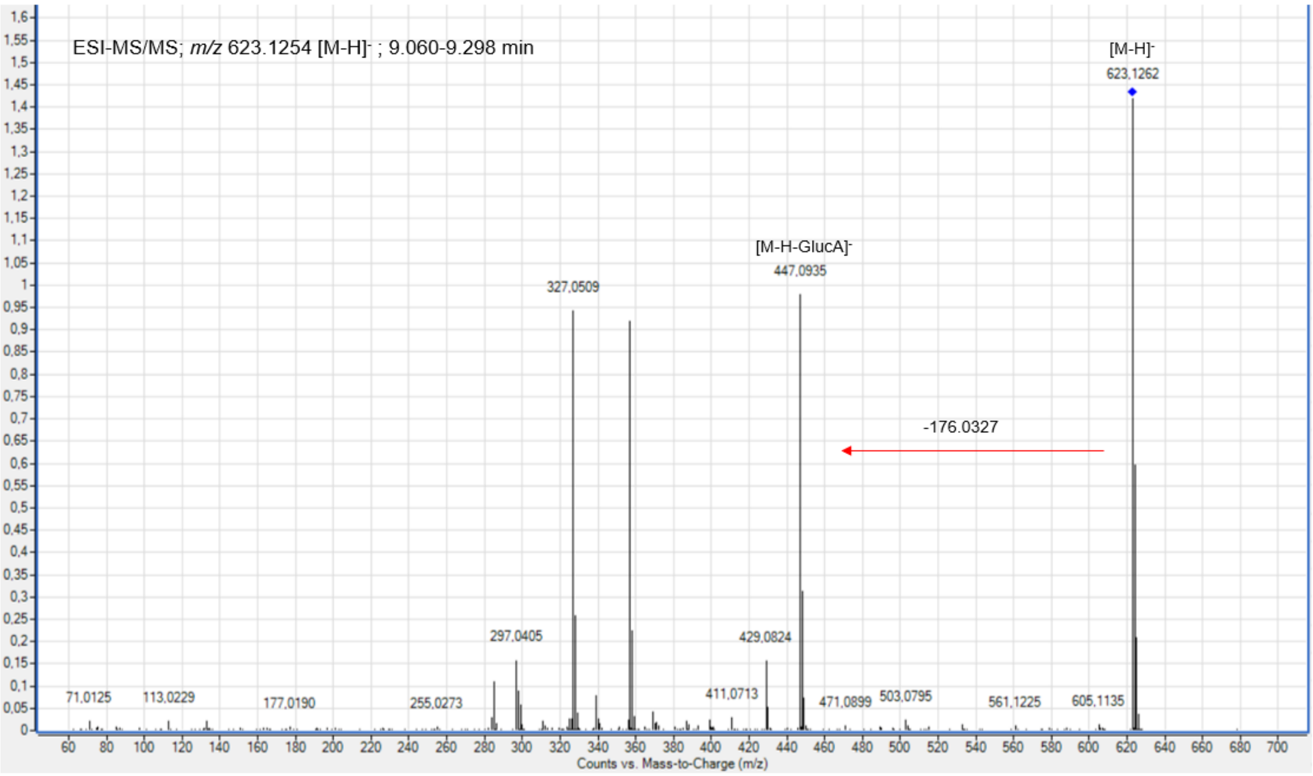

Figure S3. MS/MS spectrum of the extracted mass of isoorientin-G3 and its retention time with loss of dehydrated (-18 u) glucuronic acid (GlucA).

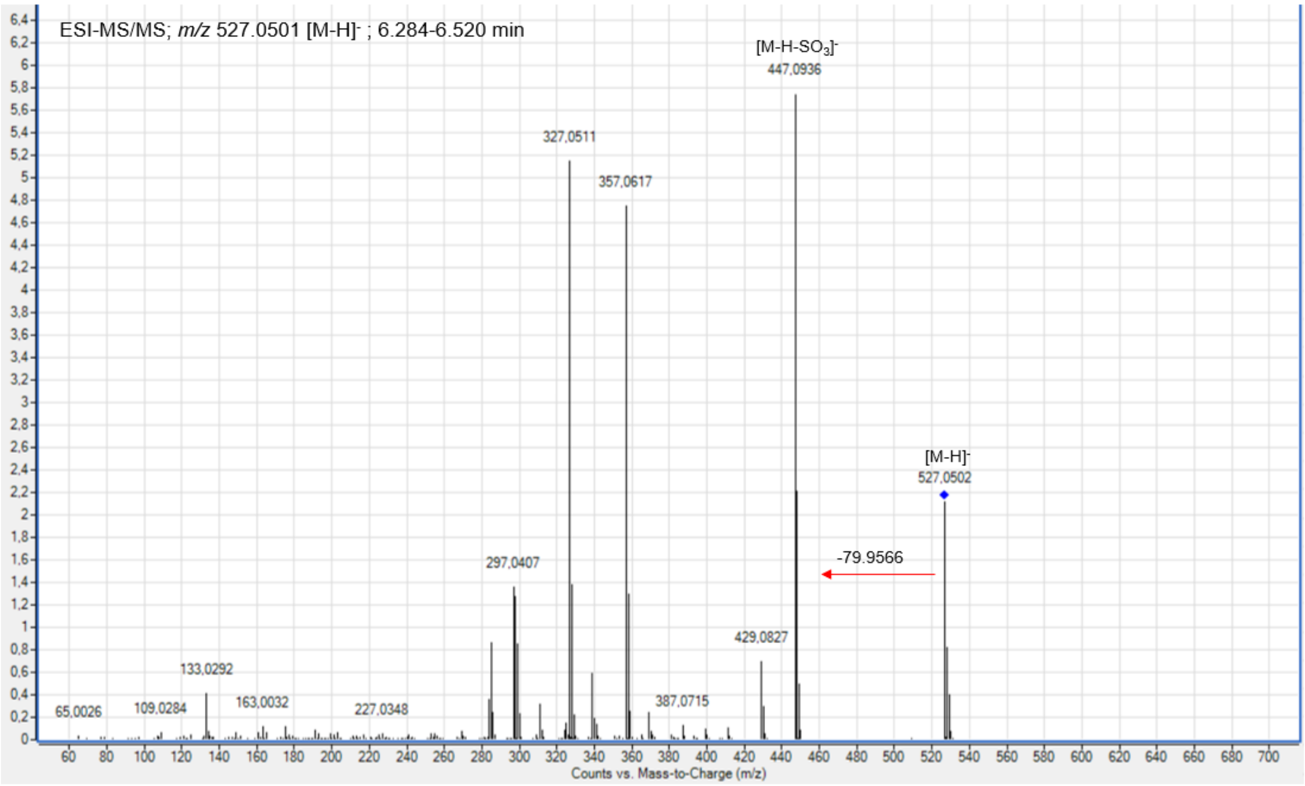

Figure S4. MS/MS spectrum of the extracted mass of isoorientin-S1 and its retention time with neutral loss of SO_3.

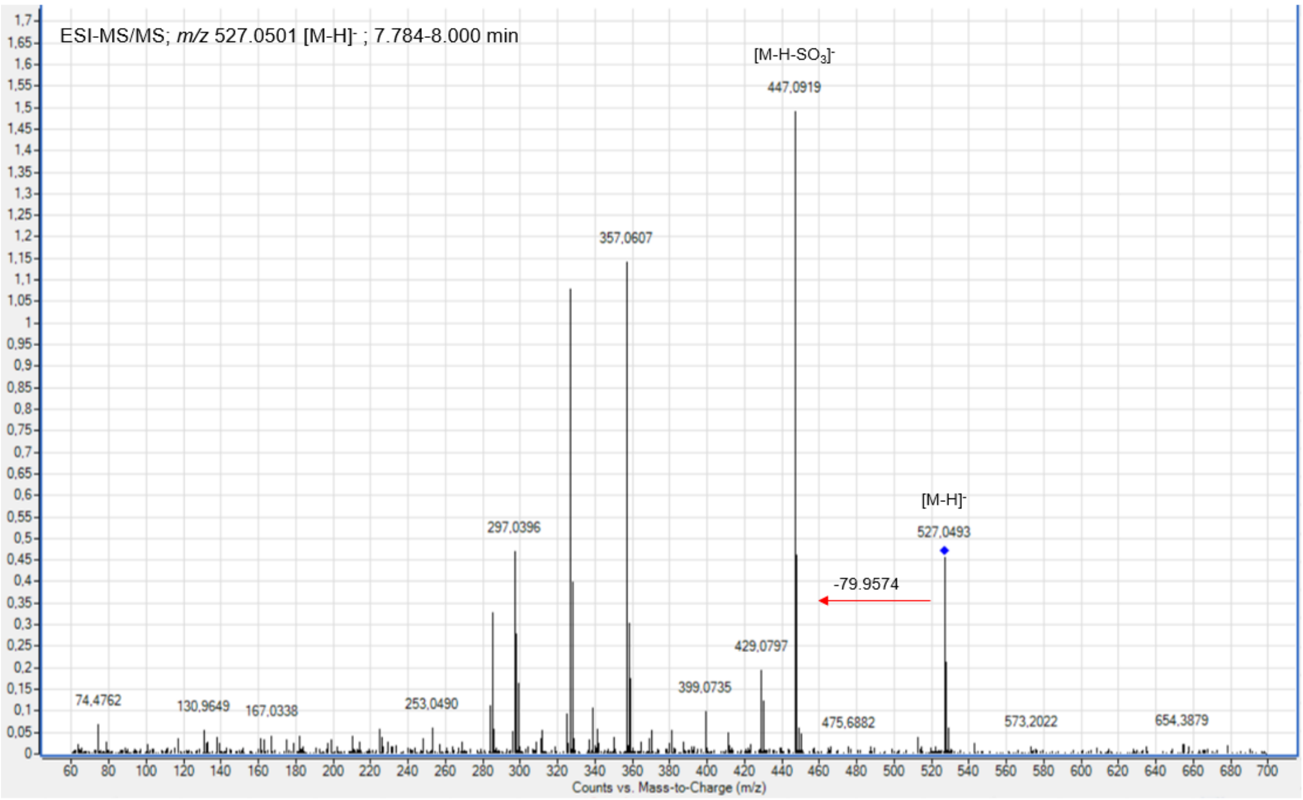

Figure S5. MS/MS spectrum of the extracted mass of isoorientin-S2 and its retention time with neutral loss of SO_3.

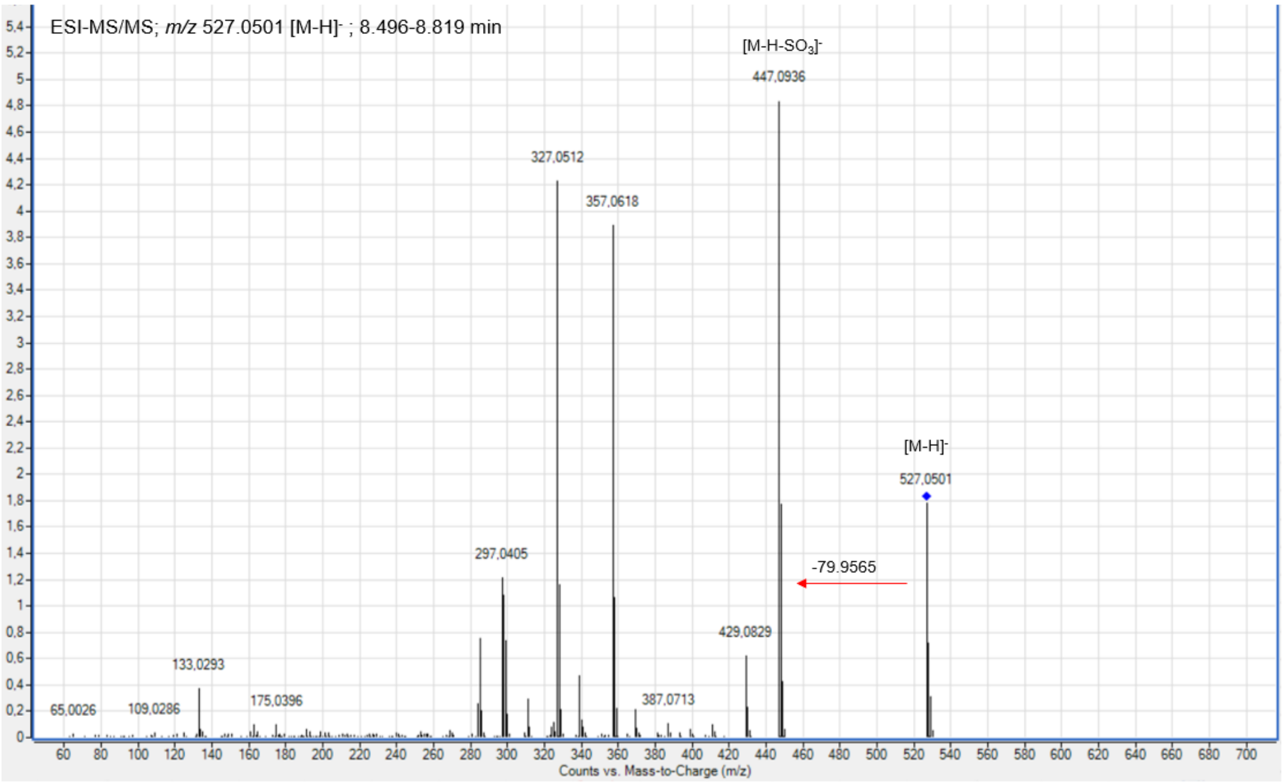

Figure S6. MS/MS spectrum of the extracted mass of isoorientin-S3 and its retention time with neutral loss of SO_3.

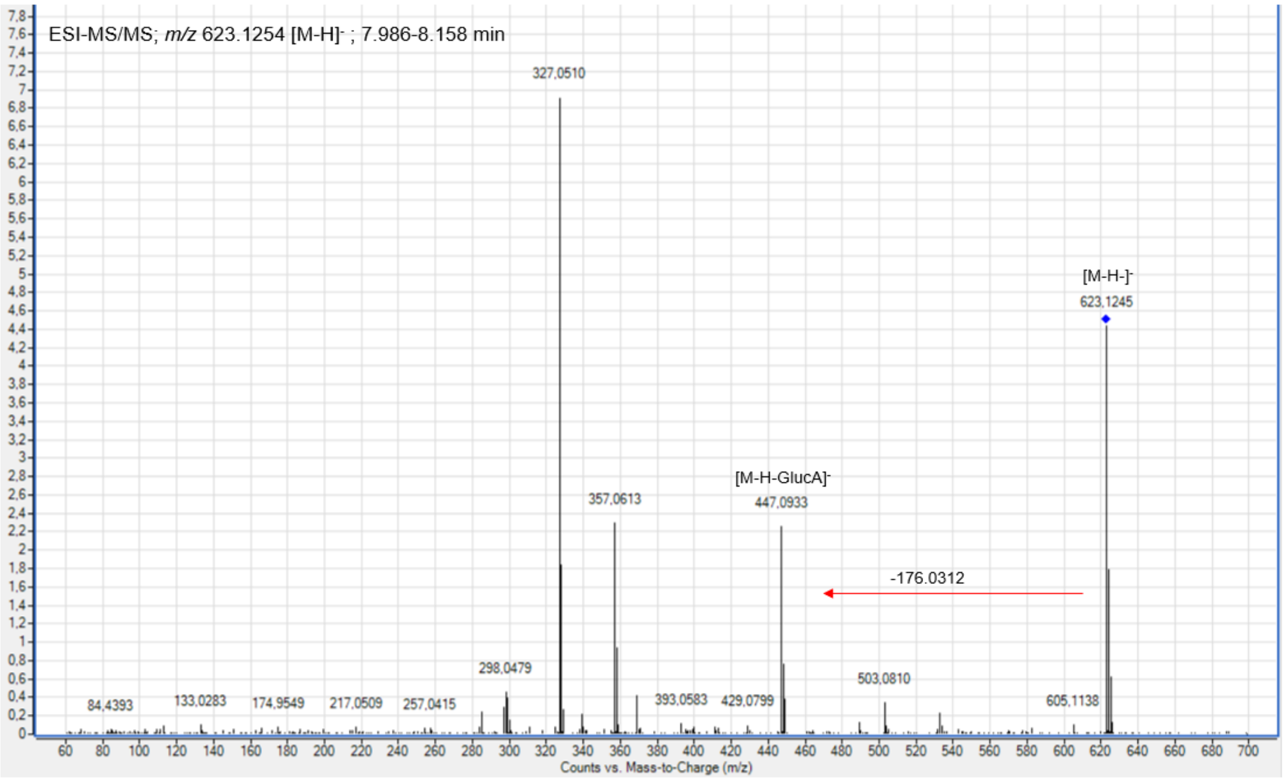

Figure S7. MS/MS spectrum of the extracted mass of orientin-G1 and its retention time with loss of dehydrated (-18 u) glucuronic acid (GlucA).

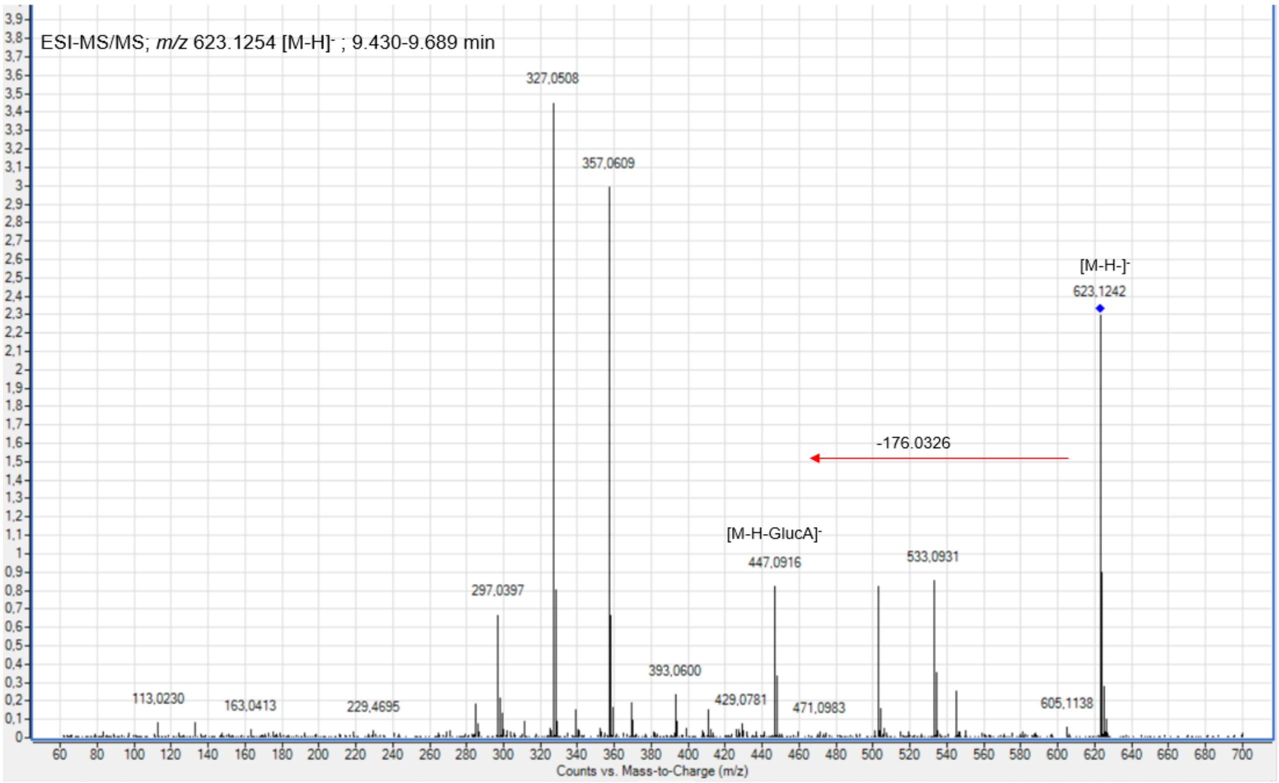

Figure S8. MS/MS spectrum of the extracted mass of orientin-G2 and its retention time with loss of dehydrated (-18 u) glucuronic acid (GlucA).

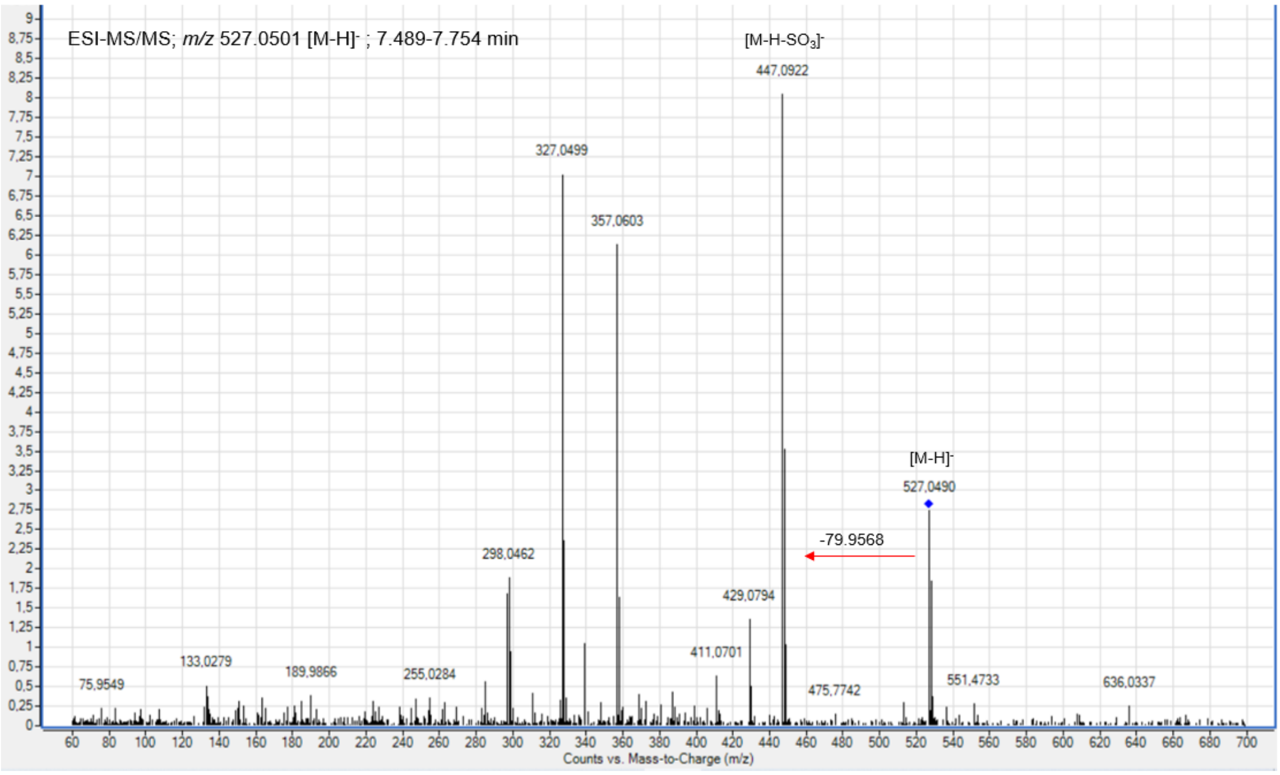

Figure S9. MS/MS spectrum of the extracted mass of orientin-S1 and its retention time with neutral loss of SO_3.

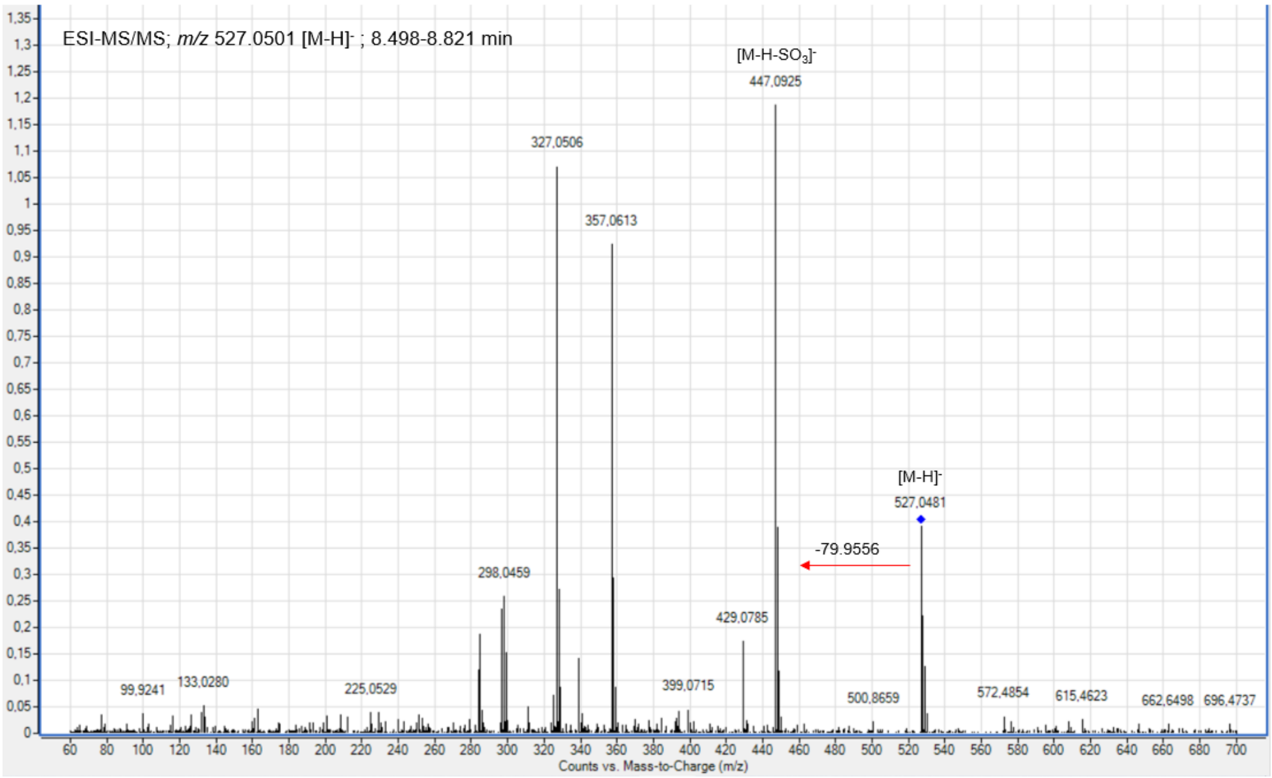

Figure S10. MS/MS spectrum of the extracted mass of orientin-S2 and its retention time with neutral loss of SO_3.

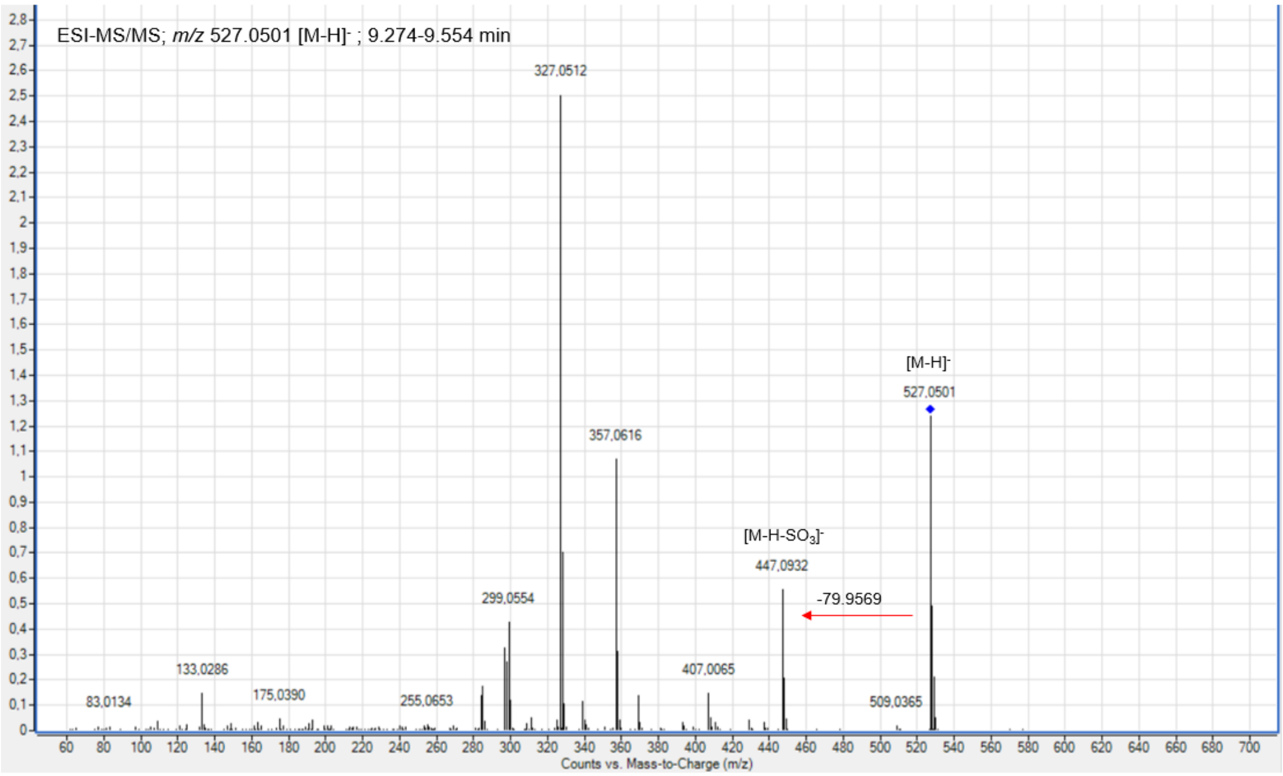

Figure S11. MS/MS spectrum of the extracted mass of orientin-S3 and its retention time with neutral loss of SO_3.

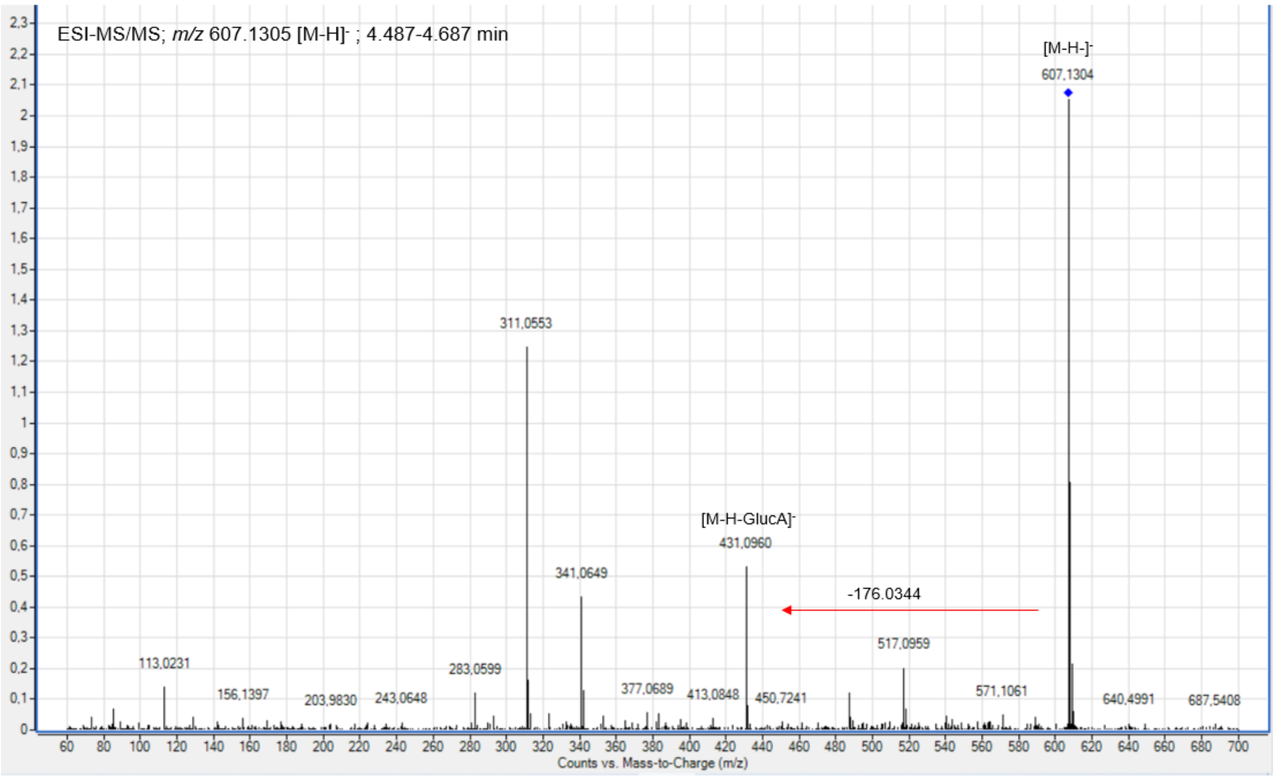

Figure S12. MS/MS spectrum of the extracted mass of vitexin-G1 and its retention time with loss of dehydrated (-18 u) glucuronic acid (GlucA).

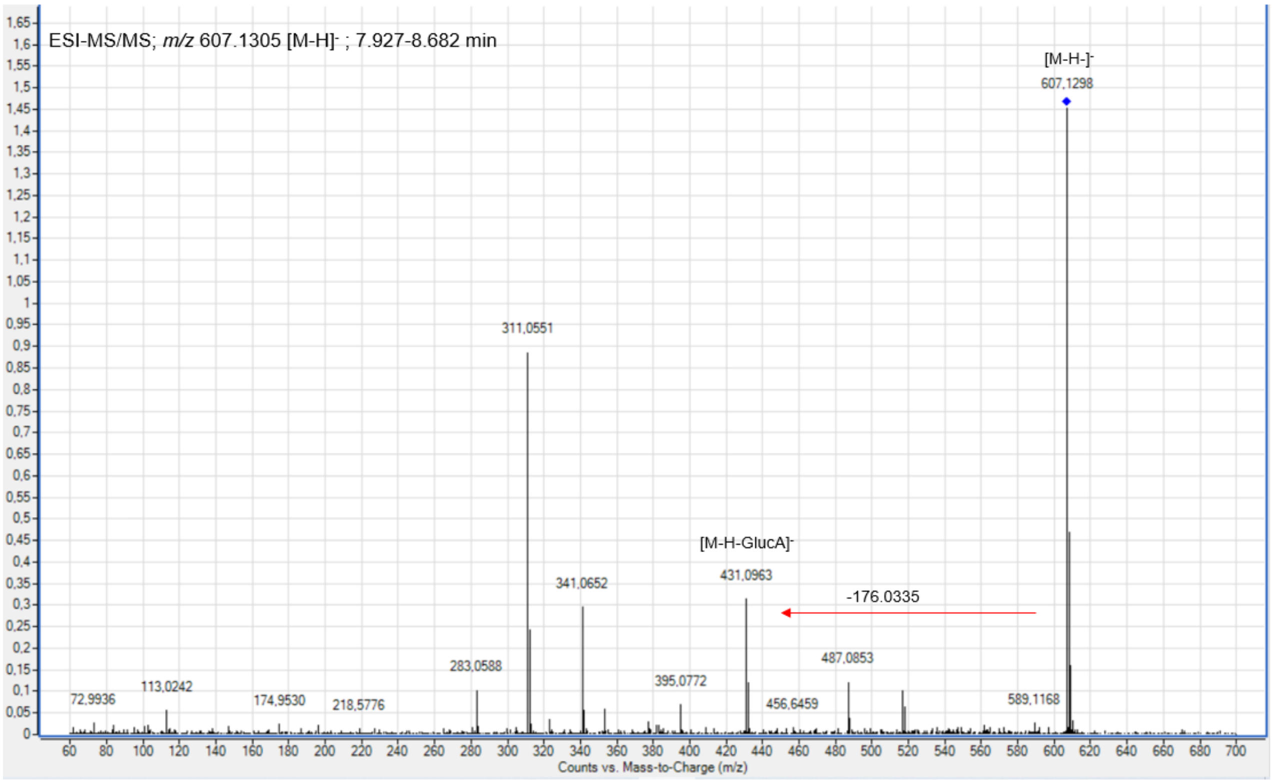

Figure S13. MS/MS spectrum of the extracted mass of vitexin-G2 and its retention time with loss of dehydrated (-18 u) glucuronic acid (GlucA).

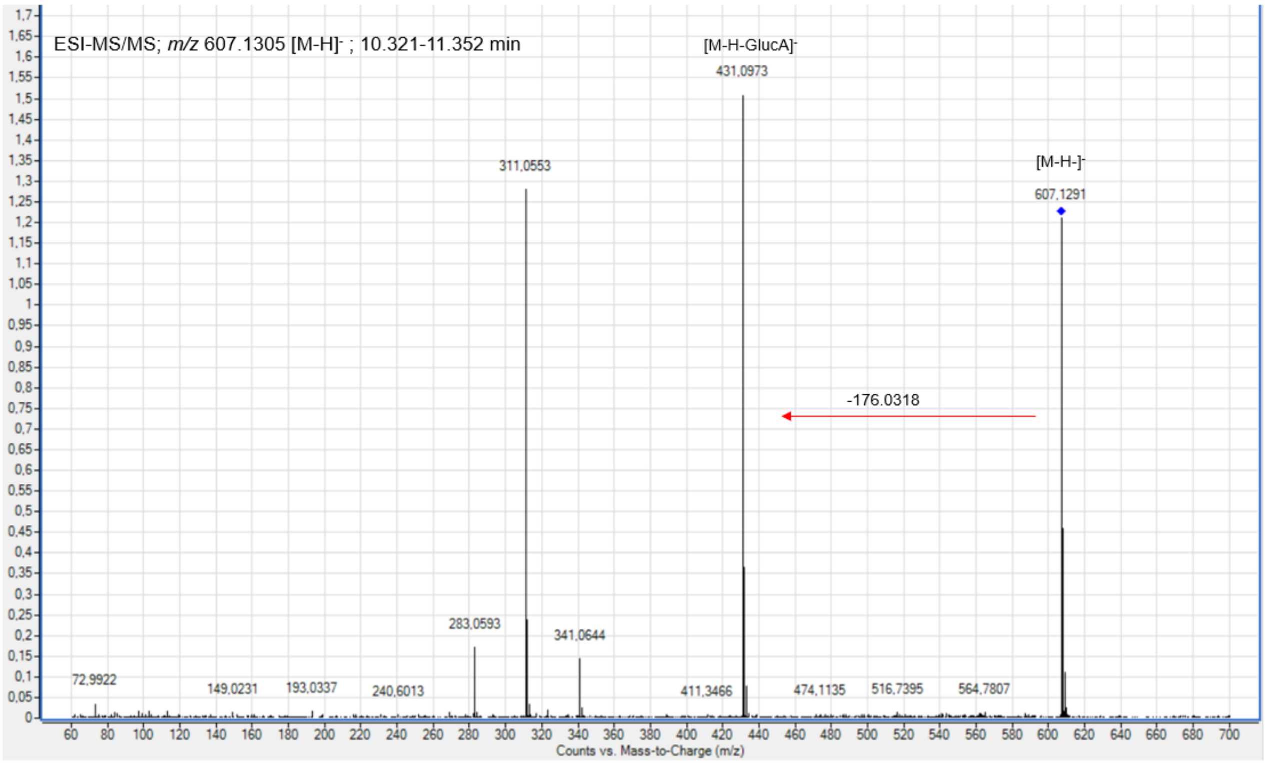

Figure S14. MS/MS spectrum of the extracted mass of vitexin-G3 and its retention time with loss of dehydrated (-18 u) glucuronic acid (GlucA).

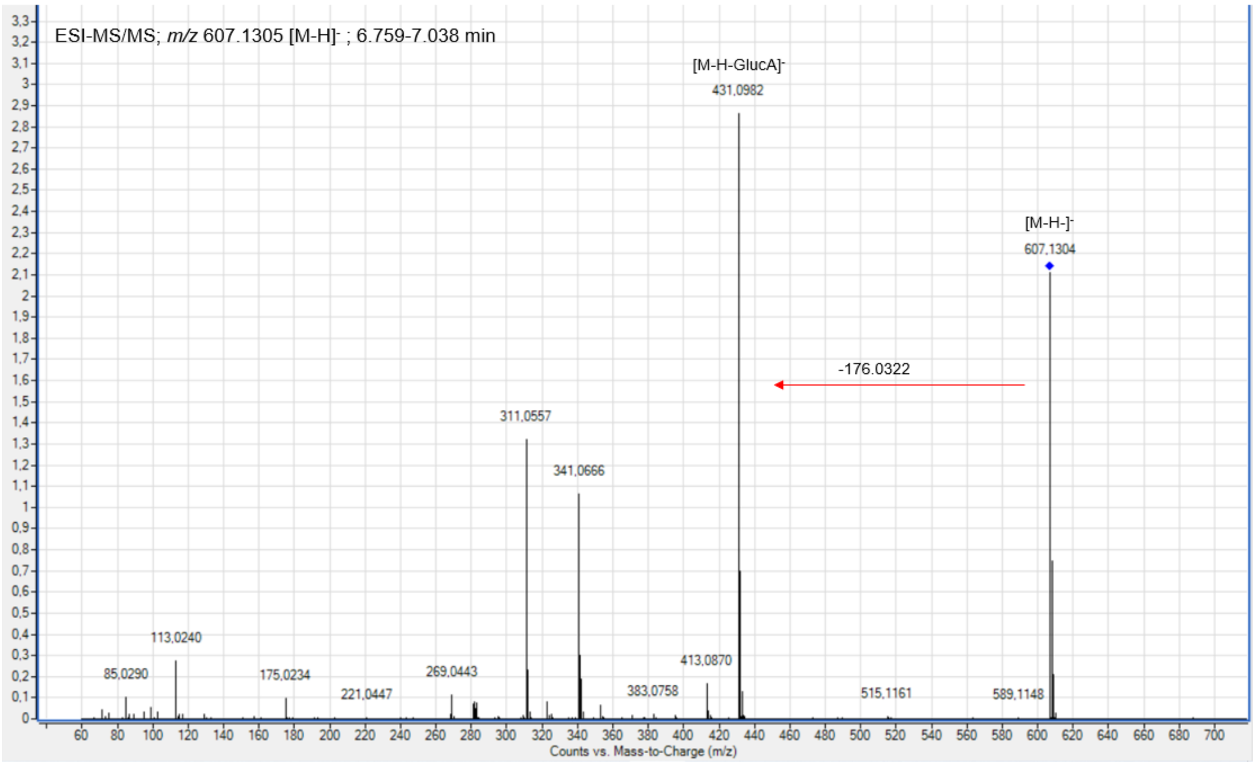

Figure S15. MS/MS spectrum of the extracted mass of isovitexin-G1 and its retention time with loss of dehydrated (-18 u) glucuronic acid (GlucA).

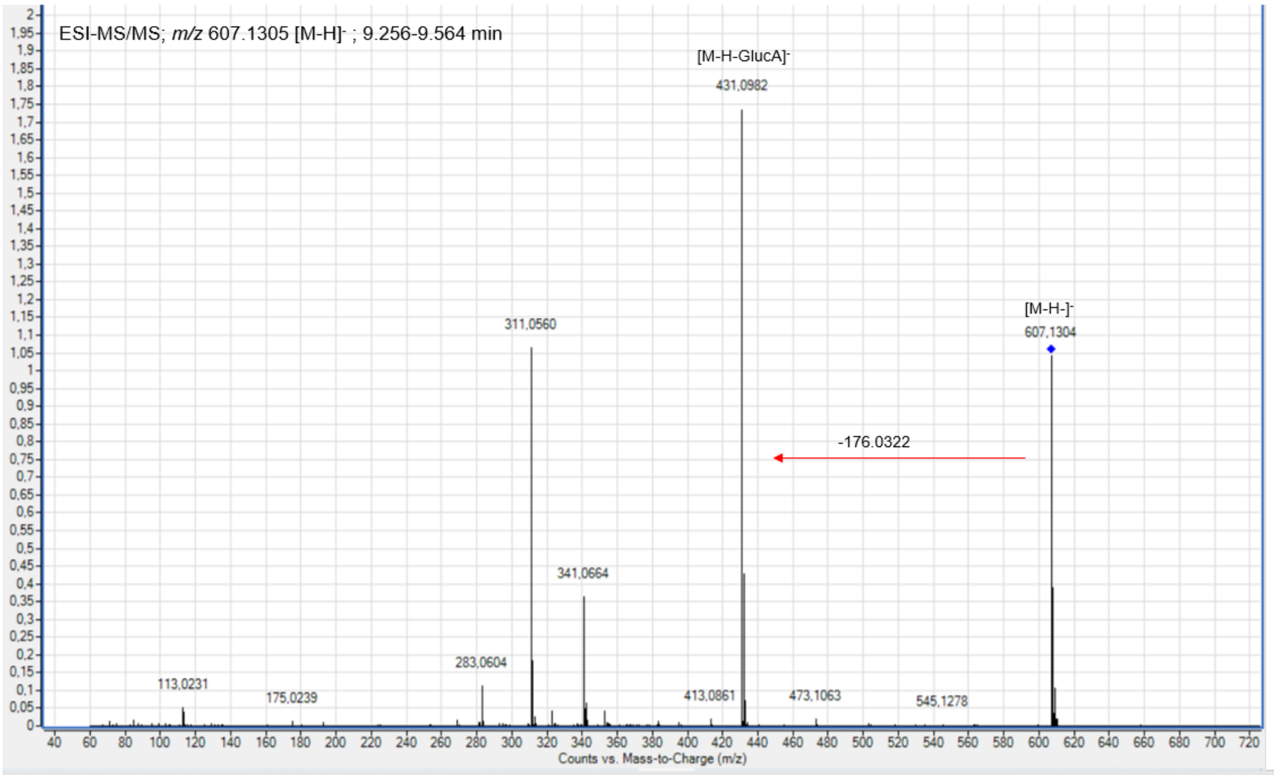

Figure S16. MS/MS spectrum of the extracted mass of isovitexin-G2 and its retention time with loss of dehydrated (-18 u) glucuronic acid (GlucA).

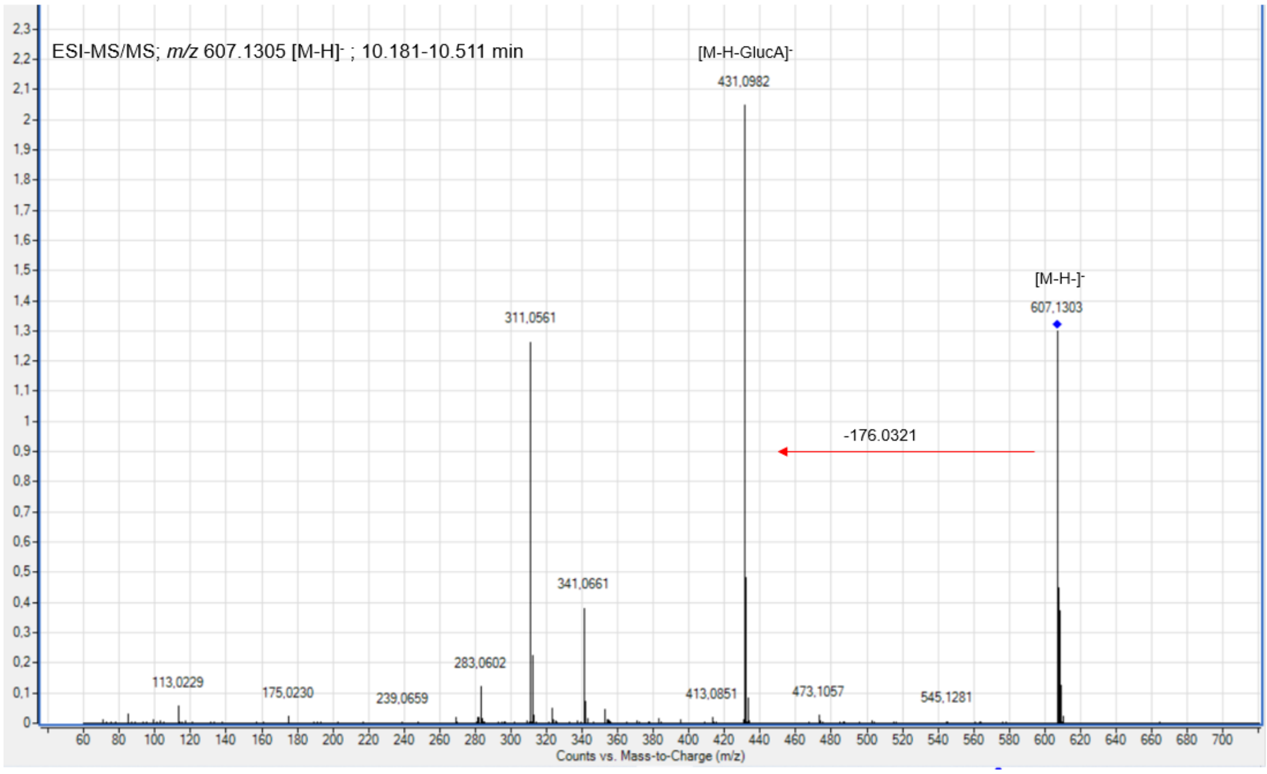

Figure S17. MS/MS spectrum of the extracted mass of isovitexin-G3 and its retention time with loss of dehydrated (-18 u) glucuronic acid (GlucA).

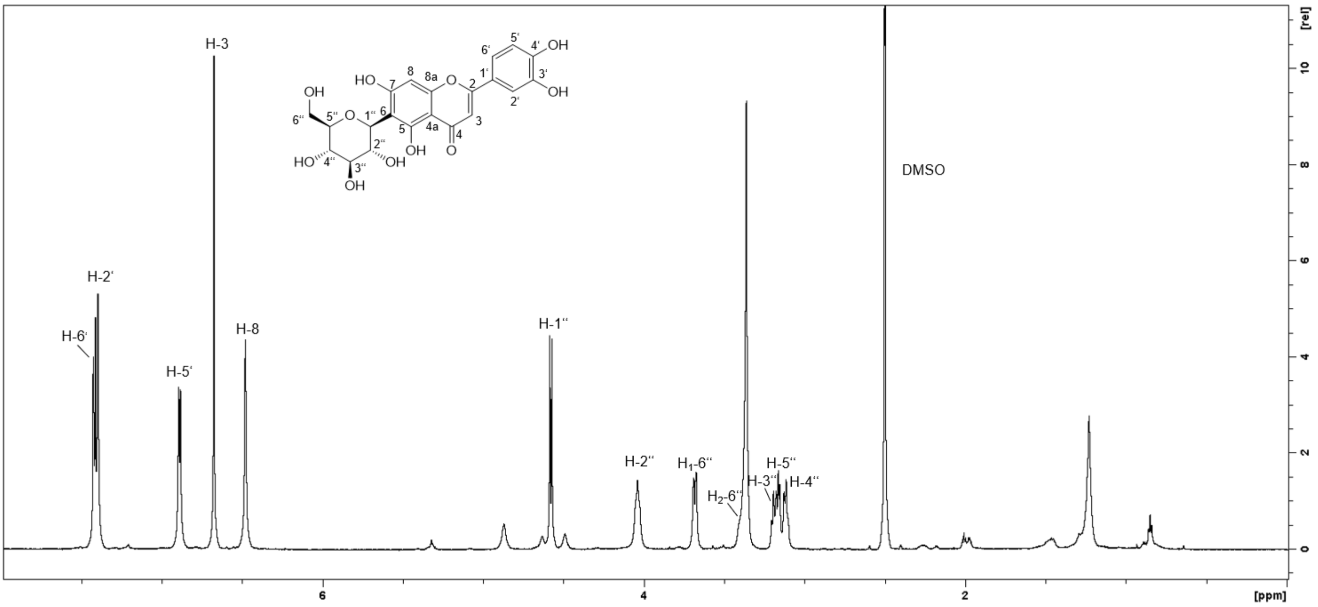

Figure S18. ^{1}H-NMR spectrum of isoorientin in DMSO-d_6 at 298 K, 700 MHz.

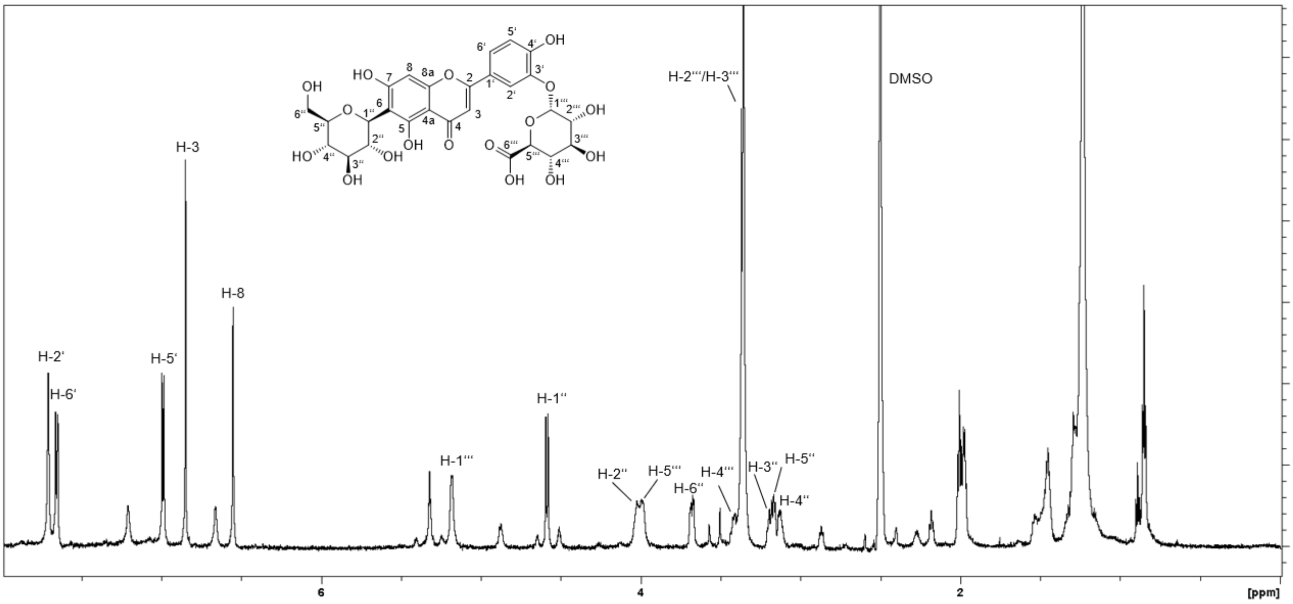

Figure S19. ^{1}H-NMR spectrum of isoorientin-3′-O-α-glucuronide in DMSO-d_6 at 298 K, 700 MHz.

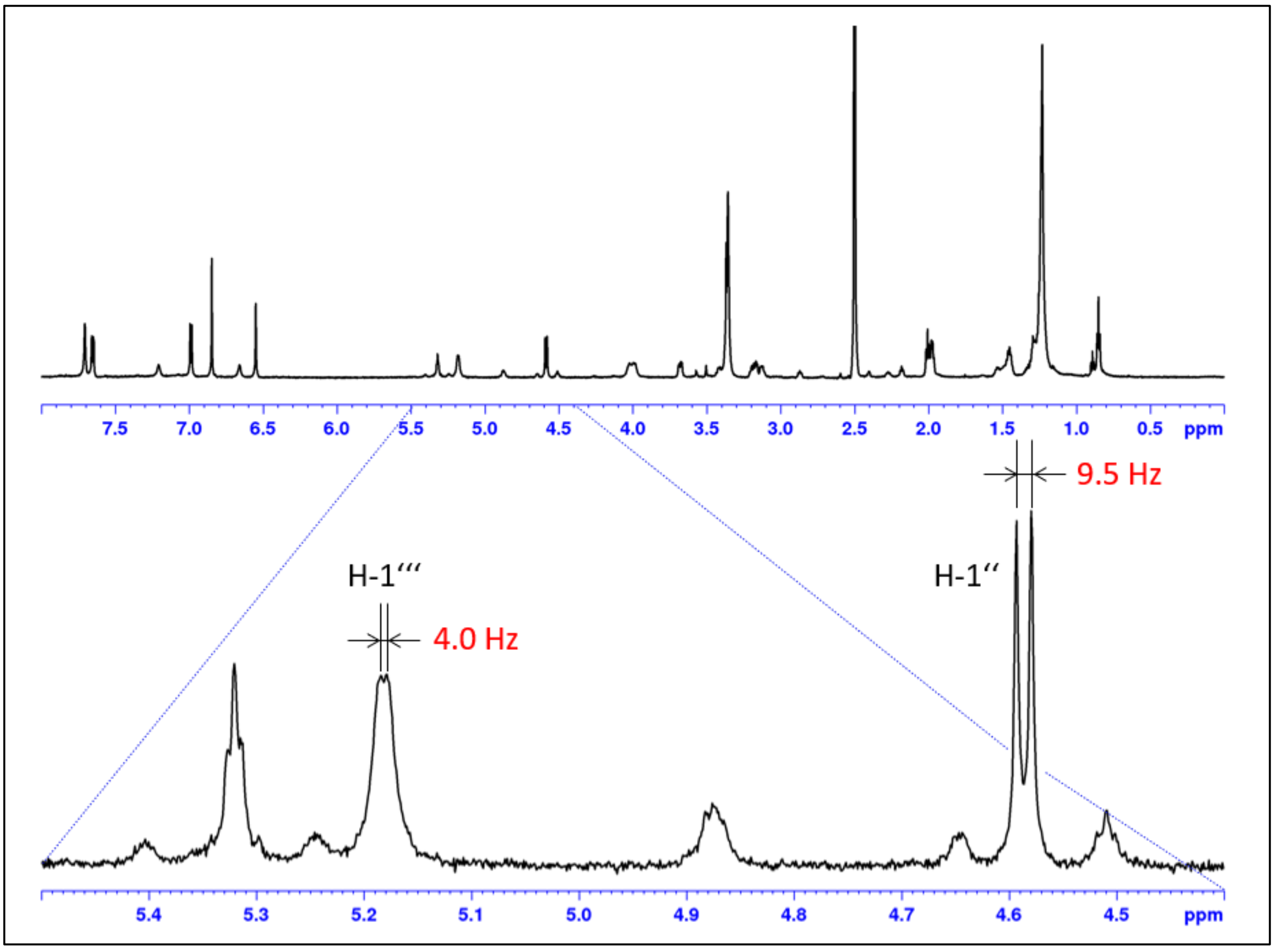

Figure S20. Expanded ^{1}H NMR spectrum of isoorientin-3′-O-α-glucuronide with coupling constants of H-1′′′ and H-1′′.

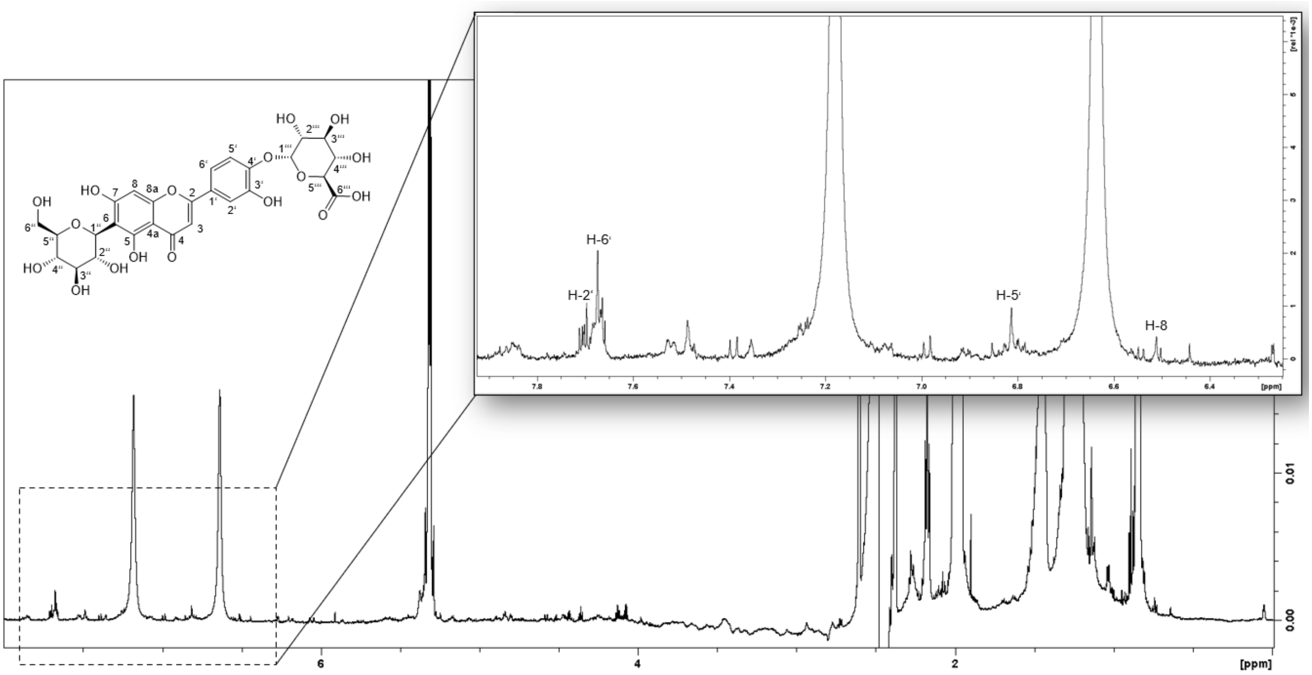

Figure S21. ^{1}H-NMR spectrum of isoorientin-4′-O-α-glucuronide in DMSO-d_6 at 298 K, 700 MHz.
